# Correlation Between Electroencephalogram Brain-to-Brain Synchronization and Team Strategies and Tools to Enhance Performance and Patient Safety Scores During Online Hexad Virtual Simulation-Based Interprofessional Education: Cross-Sectional Correlational Study

**DOI:** 10.2196/69725

**Published:** 2025-10-20

**Authors:** Atthaphon Viriyopase, Khuansiri Narajeenron

**Affiliations:** 1 Department of Emergency Medicine Faculty of Medicine Chulalongkorn University, King Chulalongkorn Memorial Hospital Bangkok Thailand

**Keywords:** brain-to-brain synchronization, EEG, electroencephalogram, emergency medicine, hyperscanning, interprofessional education, simulation, team communication, TeamSTEPPS, teamwork, virtual simulation

## Abstract

**Background:**

Team performance is crucial in crisis situations. Although the Thai version of Team Strategies and Tools to Enhance Performance and Patient Safety (TeamSTEPPS) has been validated, challenges remain due to its subjective evaluation. To date, no studies have examined the relationship between electroencephalogram (EEG) activity and team performance, as assessed by TeamSTEPPS, during virtual simulation-based interprofessional education (SIMBIE), where face-to-face communication is absent.

**Objective:**

This study aims to investigate the correlation between EEG-based brain-to-brain synchronization and TeamSTEPPS scores in multiprofessional teams participating in virtual SIMBIE sessions.

**Methods:**

This single-center study involved 90 participants (15 groups of 6 simulated professionals: 1 medical doctor, 2 nurses, 1 pharmacist, 1 medical technologist, and 1 radiological technologist). Each group completed two 30-minute virtual SIMBIE sessions focusing on team training in a crisis situation involving COVID-19 pneumonia with a difficult airway, resulting in 30 sessions in total. The TeamSTEPPS scores of each participant across 5 domains were independently assessed by 2 trained raters based on screen recording, and their average values were used. The scores of participants in the same session were aggregated to generate a group TeamSTEPPS score, representing group-level performance. EEG data were recorded using wireless EEG acquisition devices and computed for total interdependence (TI), which represents brain-to-brain synchronization. The TI values of participants in the same session were aggregated to produce a group TI, representing group-level brain-to-brain synchronization. We investigated the Pearson correlations between the TI and the scores at both the group and individual levels.

**Results:**

Interrater reliability for the TeamSTEPPS scores among 12 raters indicated good agreement on average (mean 0.73, SD 0.18; range 0.32-0.999). At the individual level, the Pearson correlations between the TI and the scores were weak and not statistically significant across all TeamSTEPPS domains (all adjusted *P*≥.05). However, strongly negative, statistically significant correlations between the group TI and the group TeamSTEPPS scores in the alpha frequency band (8-12 Hz) of the anterior brain area were found across all TeamSTEPPS domains after correcting for multiple comparisons (mean −0.87, SD 0.06; range −0.93 to −0.8).

**Conclusions:**

Strong negative correlations between the group TI and the group TeamSTEPPS scores were observed in the anterior alpha activity during online hexad virtual SIMBIE. These findings suggest that anterior alpha TI may serve as an objective metric for assessing TeamSTEPPS-based team performance.

## Introduction

The COVID-19 pandemic has highlighted the crucial importance of effective interprofessional collaboration in health care, especially in high-pressure environments such as emergency and critical care settings. Beyond individual cognitive abilities, medical professionals in these settings must work as a team, requiring shared goals, clear role understanding, and continuous communication [1]. Team Strategies and Tools to Enhance Performance and Patient Safety (TeamSTEPPS) has become a widely recognized framework for improving teamwork, communication, and overall clinical performance in such contexts. Supported by a robust body of evidence, TeamSTEPPS has been integrated into various simulation-based training programs, including online virtual simulations, which have become prominent during and after COVID-19 [2]. Although TeamSTEPPS includes 5 specific domains with validated psychometric evidence, the assessment process remains complex. Challenges include rater training, interrater reliability, and the subjective nature of evaluations. Additionally, the time and cost required for training raters add further complications [3]. Despite the availability of validated tools to measure teamwork (eg, a one-shot public goods game [4]), there are few objective measures—such as total interdependence (TI) (Multimedia Appendix 1) [5], debiased weighted phase lag index [6], and intersite phase clustering [7]—that directly link team dynamics with real-time electroencephalogram (EEG) neurological responses during collaborative tasks. An advantage of these objective measures is that they tend to predict team performance better than validated tools that are inherently subjective [8].

Hyperscanning—a method of simultaneously measuring brain activity in 2 or more individuals—has been used to explore the neural basis of social dynamics [9,10] using various neuroimaging techniques, including functional magnetic resonance imaging [11,12], functional near-infrared spectroscopy [13], EEG [14-17]. Different experimental paradigms have been used to investigate social dynamics, including movement synchronization [18], social decision-making [19], joint attention [20], team problem solving [8], team coordination [21], team communication [17], and classroom engagement [14]. Recent advancements in affordable wireless EEG technologies make EEG well suited for studying brain-to-brain synchronization, which refers to the coordinated brain activity between 2 or more individuals [22], during collaborative tasks in less controlled environments.

Reinero et al [8] demonstrated that brain-to-brain synchronization, measured by TI, predicts team performance on problem-solving tasks better than traditional self-report measures with real-time brain activity monitored using affordable wireless EEG devices. Dikker et al [14] further identified that TI-based brain-to-brain synchronization predicts class engagement and social dynamics, while Bevilacqua et al [23] extended Dikker’s findings to show that social factors, such as perceived closeness, can predict cognitive outcomes, including academic performance. These studies highlight that EEG-based brain-to-brain synchronization reflects neural alignment and provides insights into the cognitive and emotional processes underlying teamwork. However, to our knowledge, previous studies focused on face-to-face interactions, and no study has explored the correlation between EEG-based brain-to-brain synchronization and TeamSTEPPS performance in virtual simulation–based interprofessional education (SIMBIE), where face-to-face communication is absent. In additional, Guttmann et al [24] introduced stricter sample size methodologies for EEG studies, whereas Asaad and Sheth [25] emphasized the need to balance rigor and practicality, ensuring ethical and scientific standards with meaningful interpretations using efficient sample sizes. We incorporated these considerations into this study’s sample size justification to enhance research quality.

This study aims to address this gap by investigating the correlations between TI-based EEG brain-to-brain synchronization and TeamSTEPPS scores, from both individual and team perspectives, in 6 multiprofessional student groups during online virtual SIMBIE sessions. On the basis of previous studies, we hypothesized that these correlations would likely not be strongly positive owing to the absence of face-to-face communication in online virtual SIMBIE sessions. The findings may support the development of an objective, evidence-based approach to assessing team dynamics in the absence of face-to-face communication. This approach has the potential to enhance the validity and reliability of TeamSTEPPS evaluations and to facilitate automatic, real-time feedback.

## Methods

### Study Design and Setting

This study was part of a larger study [26,27] conducted at the Chulalongkorn Healthcare Advanced Multi-Profession Simulation Center in a single university hospital from August 2022 to September 2023. It was designed as a cross-sectional correlational study using quantitative approaches. The variables of interest were TeamSTEPPS scores, which measure participants’ teamwork and communication constructs of participants, and EEG features of brain activity capturing brain-to-brain synchronization; further details on TeamSTEPPS scoring are available elsewhere [28]. Although the scores and EEGs are applicable in free environments, we conducted the study during the typical hours of 6 PM to 9 PM in a controlled laboratory environment within the simulation center, where we controlled potential confounding variables (eg, temperature, visual and auditory noise, arousal confounds, motor confounds, and gameplay fluidity) [29].

This study included 30 sessions of gameplay in a virtual simulation, in which 6 participants each assumed one of 6 unique professions: (1) radiological technologist, (2) medical technologist, (3) medical doctor, (4) pharmacist, (5) circulation nurse, and (6) airway nurse. In the Emergency Room–Virtual Interprofessional Education platform—Thailand’s pioneering virtual reality (VR) system for medical interprofessional training designed and developed by Dhanakoses et al [30]—participants were represented as avatars and interacted using microphones and speakers. Multimedia Appendix 2 provides detailed descriptions of the virtually simulated scenario. Each participant attended 2 sessions; the study thus required 90 participants to complete 30 gameplay sessions. Of the 30 sessions, 10 were conducted with participants wearing VR headsets (HTC VIVE Cosmos [31]; Figure S1D in Multimedia Appendix 3), and 20 without. Participants performed cooperative tasks on their personal computers (PCs) in the laboratory. In this scenario, a male patient (aged 70 years) with chronic obstructive pulmonary disease, a history of hypertension, diabetes mellitus, and ceftriaxone allergy arrived at an emergency department with his wife. Recently discharged from an intensive care unit, his vital parameters and laboratory tests suggested hyperkalemia and COVID-19 pneumonia with acute respiratory failure. Each of the 6 participants assumed a distinct role corresponding to a fully qualified, licensed profession within the simulation scenario. The 6 simulated professions were required to collaborate to diagnose the patient and implement a treatment plan. The virtually simulated scenario emphasized the development of interprofessional communication and clinical reasoning skills and provided an ideal controlled environment to study correlations between brain-to-brain synchronization within a team and TeamSTEPPS scores.

**Figure 1:**
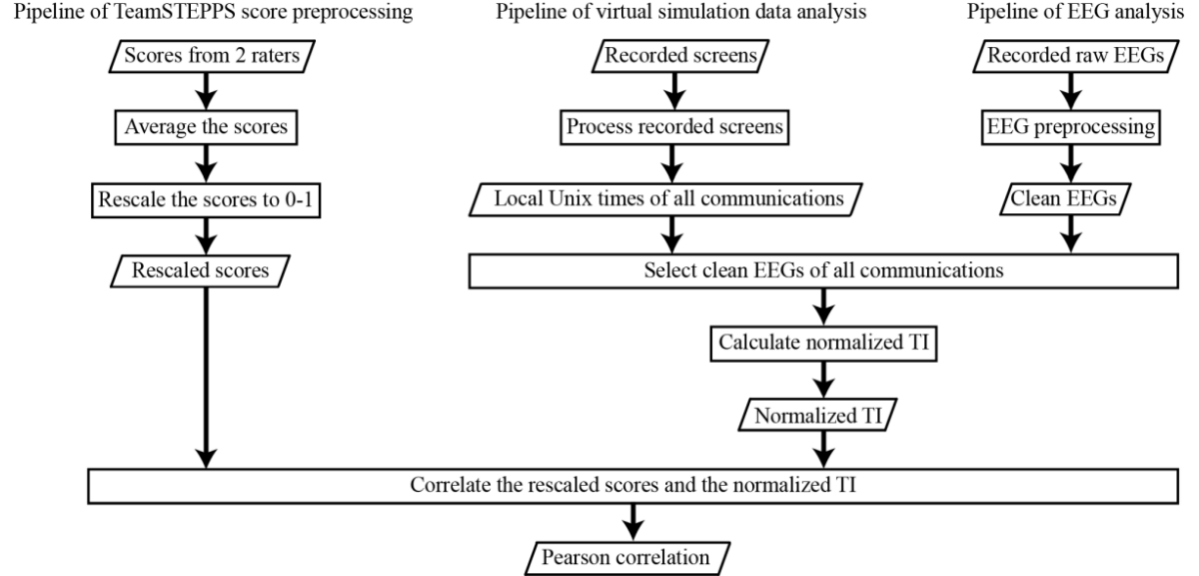
Flowchart of data analysis pipelines showing the Team Strategies and Tools to Enhance Performance and Patient Safety (TeamSTEPPS) score (left), virtual simulation data (middle), and electroencephalogram (EEG) data (right). Clean EEGs were partitioned by local Unix times of all communications, then used to calculate normalized total interdependence (TI), which was correlated with the rescaled scores.

During the sessions, we recorded each participant’s EEG signal and gameplay, while the corresponding TeamSTEPPS scores were obtained from the larger study [26]. Figure 1 provides an overview of the analysis pipelines for the 3 types of data. The EEG signal was preprocessed to mitigate artifacts, resulting in a clean EEG signal [32], while video recording of gameplay were preprocessed to extract Unix times [33] of verbal communications. The Unix times were used to segment the clean EEG signals corresponding to the communications. The segmented EEG signals were then used to compute normalized values of TI, an EEG feature measuring brain-to-brain synchronization [34]. The normalized TIs were correlated with the scaled TeamSTEPPS scores obtained by scaling the TeamSTEPPS scores to values between 0 and 1.

### Study Population

The target population comprised healthy adults aged 18-25 years who did not meet any of the following exclusion criteria: (1) predisposition to major depression, defined as a score ≥9 on the 9-item self-reported Patient Health Questionnaire-9 (PHQ-9) [35]; (2) history of substance use disorder, including cigarette smoking and alcohol addiction; (3) history of neurological or psychiatric disorders, including epilepsy; and (4) periodic use of antidepressants during the 2 weeks before the experiment. The study population, drawn from this target population, consisted of students in radiological technology, medical technology, medicine, pharmacy, and nursing from a university-affiliated hospital. We used convenience sampling by advertising the study through posters and social media on campuses, targeting 5th- or 6th-year medical students and pharmacy students, 4th-year medical technology students, as well as 3rd- or 4th-year radiological technology and nursing students. Students registered for the study through online forms without knowledge of the inclusion or exclusion criteria. Participants who were eligible and completed the experiment received monetary compensation, as indicated in the advertisement. Of 147 potential participants, 85 were eligible for the study, and 5 met the exclusion criteria but joined for practical reasons; we excluded these 5 participants from our analysis. Note that they joined the sessions without knowing that their EEG activity would be excluded from analysis. Because virtual simulators can induce simulator sickness [36], participants experiencing symptoms were administered a 10 mg oral dose of dimenhydrinate.

### Laboratory Setting

**Figure 2:**
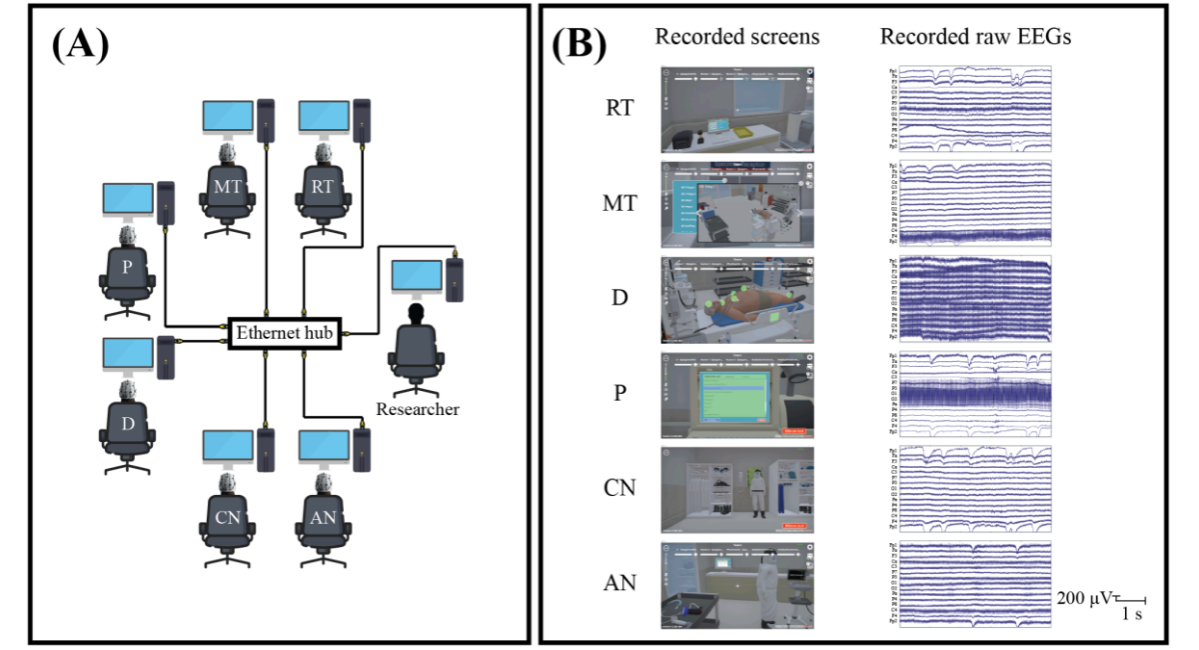
Experiment setup and recorded data. (A) 6 participants’ and the researcher’s personal computers connected in a star network topology with the ethernet hub as the center node. Collected data include participants’ video screens (B, left) and electroencephalogram (EEG) brain activity (B, right). AN: airway nurse; CN: circulation nurse; D: medical doctor; MT: medical technologist; P: pharmacist; RT: radiological technologist.

The experiments were conducted in a controlled 7×7 m room with 6 participants and a researcher, each using high-performance PCs connected through a low-latency network (Figure 2A). Non-VR sessions used monitors and audio equipment for communication, while VR sessions involved VR headsets and EEG caps, which were set up by research assistants to enhance immersion and reduce simulator sickness. The researcher’s PC managed the simulation and EEG data to ensure smooth operation (Figure 2A). Multimedia Appendix 4 provides additional details.

### Data Sources

#### TeamSTEPPS Scores

The TeamSTEPPS framework was used to assess teamwork and communication skills on a 5-point Likert scale across five topics: (1) team structure, (2) communication, (3) leadership, (4) situation monitoring, and (5) mutual support (as verified and validated in a pervious study [28]). A participant’s performance in each topic was quantitatively measured by a total score, calculated as the sum of scores ranging from 1 (worst) to 5 (best) across different aspects representing that topic. Each participant’s TeamSTEPPS scores was determined by the 5 scores from the 5 topics. The TeamSTEPPS scores for this study were obtained from the larger study [26].

#### EEG Signals

EEG signals were recorded simultaneously from 6 participants during the virtual simulation using mobile EEG devices (OpenBCI; Figure S1A in Multimedia Appendix 3) with sintered-electrode caps (Figure S1 in Multimedia Appendix 4). We used 15 passive, gel-based electrodes placed according to the international 10-20 system [37] at the following locations: Fp1, Fp2, F3, Fz, F4, C3, Cz, C4, P7, P3, Pz, P4, P8, O1, and O2 (Figure S1B in Multimedia Appendix 3). The EEG signals were initially referenced to CPz and later rereferenced to an average reference [38] during offline analysis, effectively yielding a net zero potential across the scalp for components derived from independent component analysis, thereby facilitating manual inspection. To ensure signal quality, electrode impedances were maintained below 50 kΩ on average (mean 45.59, SD 116.41 kΩ) [39], validated using OpenBCI software (version 5.1.0) [40]. EEG data was digitized at 125 Hz with high precision, and participants adjusted the sound from the virtual simulation using in-ear speakers to a comfortable level.

To monitor EEG signals in real time, we developed custom C# software that transmitted data from the participants’ PCs to the researcher’s PC (Figure S1 in Multimedia Appendix 4 provides a schematic). The process was as follows: first, EEG signals were transferred from the biosensing board of the mobile EEG device to the participant’s PC using BrainFlow software (version 4.9.0 [41]). Next, every second (125 samples), a local Unix timestamp [33] was assigned to each sample on the participant’s PC. The software then transmitted these 125-sample chunks through Ethernet using Lab Streaming Layer software (version 1.15.2 [42]) to the researcher’s PC. EEG data from all 6 participants were received and stored in a proprietary file format for offline analysis (Figure 2B, right column, shows examples of recorded EEG signals). Additionally, we performed multiple sanity checks to ensure that the data received by the researcher met strict quality standards (Multimedia Appendix 5).

#### Video Recordings of Virtually Simulated Scenarios

**Figure 3:**
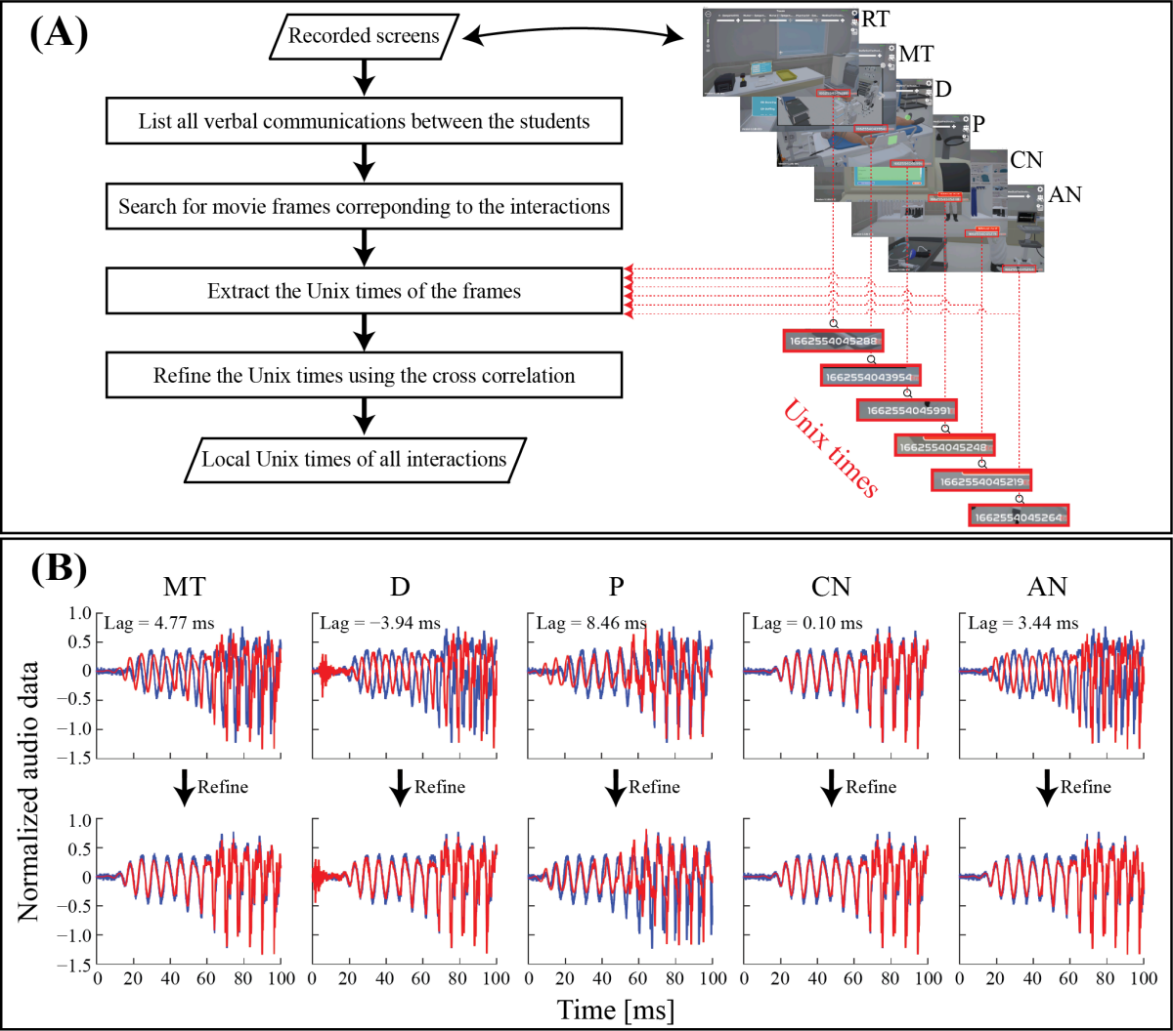
Temporal alignment of verbal social interactions. (A) Extraction of temporal information from recorded screens, indicated by Unix times in red boxes. (B) Adjustment of time lags between participants’ audio using cross-correlation, with RT as reference (blue) and other participants (red) before (top) and after (bottom) compensation. AN: airway nurse; CN: circulation nurse; D: medical doctor; MT: medical technologist; P: pharmacist; RT: radiological technologist.

Participants’ PC screens during the virtually simulated scenario were recorded at 60 frames per second with a resolution of 2560×1440 pixels, and audio was captured at 48,000 Hz using NVIDIA ShadowPlay software (version 3.25.1.27 [43]; Figure 2B, left column, shows examples of the 6 PC screens). In addition to capturing participants’ actions, the screen recordings displayed local Unix timestamps [33], which were obtained using the DateTime function of C# to precisely time each action (Figure 3A, red boxes, shows examples). These video and audio recordings were saved locally on each participant’s PC in MP4 format. The recorded screen footage was later used for offline synchronization between the simulated scenario actions and corresponding EEG signals.

### Data Preparation

#### Preprocessing of the TeamSTEPPS Scores

Two trained raters independently rated the TeamSTEPPS scores for each profession (Table 1, “Rater 1” and “Rater 2” rows [26]). We averaged the scores from the 2 raters and scaled them using the minimum and maximum values (Table 1, “Range” rows), resulting in scaled TeamSTEPPS scores with values between 0 and 1 (Table 1, “Scaled AVG” rows). Additionally, we created an overall TeamSTEPPS score by summing all 5 topic scores to summarize each participant’s teamwork and communication skills (Table 1, “Overall” column). Analogously, we defined a scaled overall TeamSTEPPS score (Table 1, “Scaled AVG” rows intersecting the “Overall” column). We evaluated the performance of a team using a group TeamSTEPPS score, calculated by averaging the scaled overall TeamSTEPPS scores of all 6 professions in the team.

**Table 1:** Descriptive statistics of the Team Strategies and Tools to Enhance Performance and Patient Safety (TeamSTEPPS) scores (N=30).

Professions				TeamSTEPPS scores																																								
				Team structure							Communication							Leadership							Situation monitoring							Mutual support							Overall					
**Radiological technologist (n=22)**																																												
	Range								2-10							4-20							5-25							5-25							4-20							20-100
	Rater 1, mean (SD)								5.86 (2.88)							8.45 (2.65)							10.73 (2.35)							12.5 (2.52)							6.27 (1.55)							43.82 (9.73)
	Rater 2, mean (SD)								5.64 (3.03)							9.27 (2.45)							10.95 (3.43)							12.59 (2.36)							5.77 (1.15)							44.23 (10.44)
	ICC^a^								0.94							0.74							0.73							0.63							0.35							0.89
	Scaled AVG^b^, mean (SD)								0.47 (0.36)							0.30 (0.15)							0.29 (0.14)							0.38 (0.11)							0.13 (0.07)							0.30 (0.12)
**Medical technologist (n=30)**																																												
	Range					2-10							4-20							5-25							4-20							3-15							18-90			
	Rater 1, mean (SD)					5.87 (3.34)							10.23 (3.26)							13.4 (4.38)							11.73 (2.05)							5.70 (3.11)							46.93 (12.74)			
	Rater 2, mean (SD)					5.87 (3.35)							10.00 (3.36)							13.43 (4.40)							11.67 (2.12)							5.40 (2.77)							46.3 (12.54)			
	ICC					>0.99							0.96							>0.99							0.99							0.94							0.99			
	Scaled AVG, mean (SD)					0.48 (0.42)							0.38 (0.20)							0.42 (0.22)							0.48 (0.13)							0.21 (0.24)							0.40 (0.18)			
**Medical doctor (n=30)**																																												
	Range				4-20							4-20							6-30							5-25							4-20							23-115				
	Rater 1, mean (SD)				12.23 (4.62)							13.13 (4.24)							17.43 (5.68)							16.80 (3.47)							10.33 (4.41)							69.93 (20.53)				
	Rater 2, mean (SD)				14.07 (2.99)							13.63 (3.77)							21.30 (5.51)							16.67 (3.54)							11.07 (3.41)							76.73 (17.71)				
	ICC				0.64							0.76							0.64							0.61							0.73							0.81				
	Scaled AVG, mean (SD)				0.57 (0.22)							0.59 (0.24)							0.56 (0.22)							0.59 (0.16)							0.42 (0.23)							0.55 (0.20)				
**Pharmacist (n=30)**																																												
	Range						2-10							4-20							5-25							2-10							4-20							17-85		
	Rater 1, mean (SD)						5.90 (3.14)							15.07 (3.95)							18.73 (4.29)							6.10 (2.16)							11.57 (3.88)							57.37 (13.88)		
	Rater 2, mean (SD)						4.77 (3.51)							13.90 (3.66)							15.33 (3.84)							6.97 (2.65)							8.43 (3.49)							49.40 (14.13)		
	ICC						0.78							0.56							0.66							0.43							0.51							0.75		
	Scaled AVG, mean (SD)						0.42 (0.40)							0.66 (0.21)							0.60 (0.20)							0.57 (0.26)							0.38 (0.21)							0.54 (0.20)		
**Circulation nurse (n=28)**																																												
	Range					3-15							4-20							5-25							5-25							4-20							21-105			
	Rater 1, mean (SD)					9.14 (3.76)							11.75 (3.45)							13.32 (3.67)							12.71 (2.57)							6.43 (2.18)							53.36 (12.89)			
	Rater 2, mean (SD)					8.71 (3.49)							11.82 (4.11)							12.54 (3.43)							11.75 (2.03)							5.96 (1.40)							50.79 (11.81)			
	ICC					0.89							0.84							0.65							0.66							0.32							0.92			
	Scaled AVG, mean (SD)					0.49 (0.29)							0.49 (0.23)							0.40 (0.17)							0.36 (0.11)							0.14 (0.09)							0.37 (0.15)			
**Airway nurse (n=30)**																																												
	Range							3-15							4-20							5-25							5-25							4-20							21-105	
	Rater 1, mean (SD)							9.13 (4.22)							11.43 (4.20)							15.80 (5.17)							16.37 (4.79)							11.63 (4.32)							64.37 (21.36)	
	Rater 2, mean (SD)							8.30 (3.68)							11.63 (3.55)							15.97 (3.70)							16.27 (3.51)							12.33 (4.18)							64.50 (16.26)	
	ICC							0.89							0.77							0.81							0.73							0.75							0.88	
	Scaled AVG, mean (SD)							0.48 (0.32)							0.47 (0.23)							0.54 (0.21)							0.57 (0.19)							0.50 (0.25)							0.52 (0.22)	
**Group (n=20)**																																												
	Scaled AVG, mean (SD)						0.49 (0.32)							0.49 (0.19)							0.47 (0.17)							0.50 (0.12)							0.31 (0.15)							0.45 (0.17)		
**Complete group (n=9)**																																												
		Scaled AVG, mean (SD)	0.50 (0.40)							0.47 (0.23)							0.47 (0.22)							0.49 (0.13)							0.31 (0.18)							0.45 (0.21)						

^a^ICC: intraclass correlation coefficient.

^b^Scaled AVG: scaled average.

#### EEG Preprocessing

Overall, EEG preprocessing removed noise, corrected artifacts, rereferenced signals, and reduced dimensionality. Cleaned EEG data were filtered (1-40 Hz), grouped into 3 brain regions, and prepared for independent component analysis and TI analysis to ensure high-quality results (details provided in Multimedia Appendix 6).

#### Preprocessing of the Video Recordings: Aligning EEG Signals With the Recordings

To conduct our hyperscanning study in the virtual simulation, we focused on synchronizing verbal social interactions recorded from participants’ screens. Figure 3A (left side) illustrates the flowchart for processing these recordings to obtain Unix times corresponding to the interactions. First, we imported the recordings of 6 participants from the same session into Adobe Premiere Pro (version 22.2.0) and marked the start and end frames of each verbal interaction by visually matching the auditory stimuli of both the sender and receiver (Figure 3B, top row). After identifying these frames, we extracted the corresponding Unix timestamps (Figure 3A, red boxes). To enhance the accuracy of the timestamps, we adjusted for any lag using cross-correlation analysis of the average auditory stimuli from both left and right channels (Figure 3B, bottom row). This refined timing was then aligned with the clean EEG signal timestamps, allowing us to isolate periods of EEG data associated with specific verbal interactions. These selected time periods were crucial for calculating brain-to-brain synchronization among participants.

#### Computation of Normalized TI

In this study, we used the TI method to assess brain-to-brain synchronization among participants during the virtual simulation. TI is a technique that analyzes the temporal relationships between two signals, extending beyond zero-lag [34]. For our analysis, we focused on 2 specific frequency ranges: (1) all frequencies between 1-20 Hz defined as all frequency bands; and (2) the alpha frequency band (8-12 Hz), given its putative role in attention regulation [44,45] and cognitive control [46,47]. Further mathematical details regarding the TI calculation can be found in Multimedia Appendix 1.

**Figure 4:**
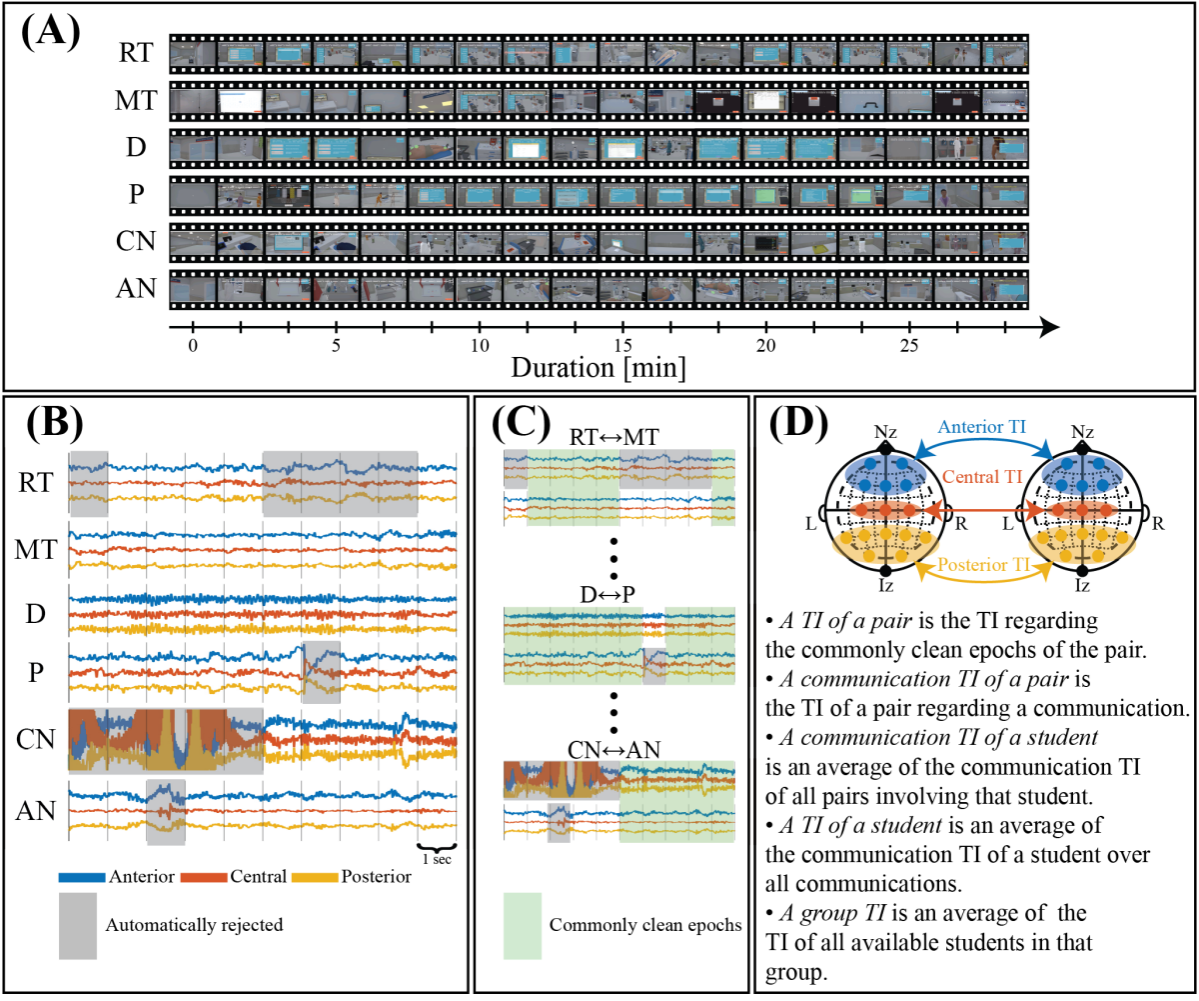
Steps for calculating total interdependence (TI). (A) Alignment of recorded screens from 6 participants during a simulated scenario. (B) Aligned clean electroencephalogram (EEG) signals epoched into 1-second periods (vertical bars), with rejected epochs (gray boxes) exceeding 6 times the golden SDs. (C) Commonly clean epochs (green) for participant pairs, used to calculate TIs across anterior (blue), central (red), and posterior (yellow) brain areas (D). AN: airway nurse; CN: circulation nurse; D: medical doctor; Iz: Inion; L: left; MT: medical technologist; Nz: Nasion; P: pharmacist; R: right; RT: radiological technologist.

TI calculation involved aligning participants’ recorded screens (Figure 4A) and segmenting EEG signals into 1-second epochs (Figure 4B). Epochs exceeding 6 times the golden SDs—nonbiased estimates of variability derived from clean EEG signals (Multimedia Appendix 6)—were rejected (Figure 4B, gray boxes). The remaining commonly clean epochs (Figure 4C, green boxes) were used to compute TIs for the anterior, central, and posterior brain regions (Figure 4D). Because TI depends on duration, normalization was necessary (Multimedia Appendix 1 provides details on normalizing TI). We applied this procedure to calculate the communication TI for all possible pairs of participants. We defined the communication TI of a student as the average of the communication TIs of all pairs including that participant, and the student TI as the average of the communication TI across all valid communications for that student. We then derived a group TI by averaging all student TIs in that group. TIs were computed separately for the anterior, central, and posterior brain regions (Figure 4D). To our knowledge, this is the first study to apply the TI normalization technique.

### Statistical Analysis

Because the TeamSTEPPS scores for each profession were independently assessed by 2 trained raters, we evaluated interrater reliability using the intraclass correlation coefficient (ICC; [48]). We selected a 2-way random-effects model, which assumes that both raters and participants were randomly chosen, to generalize the reliability estimates to any trained raters. Given the resource-intensive nature of rater training, we were focused on estimating the reliability of scores if only one rater was used. Accordingly, we adopted the “single rater” type and the “absolute agreement” definition, following the reporting guidelines proposed by Koo and Li [49]. The ICC was calculated using the *icc* function from the *irr* package (version 0.84.1 [50]) in R software (version 4.3.2; R Foundation for Statistical Computing), with the selected model, type, and definition options, while other parameters were set to default. To aid interpretation of ICC values, we applied Cicchetti’s [51] thresholds, categorizing reliability as excellent (ICC=0.75-1.00), good (0.60-0.74), fair (0.40-0.59), and poor (<0.40). We also note that Koo and Li [49] proposed an alternative system in which ICC values <0.5, 0.5-0.75, 0.75-0.9, and >0.9 correspond to poor, moderate, good, and excellent reliability, respectively.

Since the study involved 2 variables (TeamSTEPPS and TI), analyses were performed with multivariate outliers, which were identified using the Minimum Covariance Determinant [52] using the *MASS* package (version 7.3.60.0.1 [53]) in R software (version 4.3.2) with 75% of the samples regarded as the minimum number of “good” samples and an α level of .001. Correlation between both variables was assessed using the Pearson correlation for 36 cases: a total of 6 for the TeamSTEPPS scores (5 topics and 1 overall), 3 for the brain areas (anterior, central, and posterior), and 2 for the frequency bands (all frequency bands and the alpha frequency band). To control for Type I error, the Benjamini and Hochberg procedure [54,55] was applied for multiple comparison with a combined threshold of *P*<.05.

### Sample Size Justification

As part of the larger study [26,27], our sample size of 30 sessions aligned with Reinero’s [8] study, which included a total of 44 groups. A total of 30 sessions in this study yielded 180 observations (90 participants × 2 sessions), which is comparable to the 176 observations (4 participants × 44 groups) reported in Reinero’s [8] study. With a desired statistical power of 0.8 and a sample size of 30 sessions, we calculated the minimum detectable effect size for correlation to be 0.49 using the formula provided elsewhere [56].

### Ethical Considerations

The study was approved by the Institutional Review Board of the Faculty of Medicine at Chulalongkorn University (COA No 1085/2022). All participants provided written informed consent to participate. Participants who completed the experiment received THB 1000 (US $30.27).

## Results

### Demographic

We included a total of 85 students, with a mean age of 21.87 (SD 1.17) years, a mean grade point average of 3.21 (SD 0.38), and a mean PHQ-9 score of 3.49 (SD 2.54). Of these, there were 11 radiological technologists, 15 medical technologists, 15 medical doctors, 15 pharmacists, 14 circulation nurses, and 15 airway nurses. A total of 20 sessions had complete EEG activities of all 6 simulated professions. Table 2 presents the unweighted demographic characteristics of the included participants for each simulated profession. Of the 59 women (69% of the total sample), 8 (14%) were radiological technologists, 12 (20%) were medical technologists, 4 (7%) were medical doctors; 7 (12%) were pharmacists; 13 (22%) were circulation nurses; and 15 (25%) were airway nurses. A total of 13 (15%) students were administered a 10 mg dose of oral dimenhydrinate.

**Table 2:** Demographic characteristics.

Characteristics		Professions (N=85)																									
		Total				Radiological technologist (n=11)			Medical technologist (n=15)				Medical doctor (n=15)				Pharmacist (n=15)			Circulation nurse (n=14)			Airway nurse (n=15)				
Age (years), mean (SD)		21.87 (1.17)				21.36 (0.50)			21.27 (0.46)				22.47 (0.74)				23.47 (1.19)			21.36 (1.01)				21.13 (0.64)			
**Sex, n**																											
	Female		59				8			12				4				7			13				15		
	Male		26				3			3				11				8			1				—^a^		
**Academic year**																											
	3			13			—				—				—			—			6					7	
	4			44			11				15				—			2			8					8	
	5			18			—				—				12			6			—					—	
	6			10			—				—				3			7			—					—	
Grade point average, mean (SD)				3.21 (0.38)			3.23 (0.4)				3.11 (0.38)				3.34 (0.44)			3.20 (0.46)			3.21 (0.27)					3.19 (0.31)	
PHQ-9^b^ score, mean (SD)					3.49 (2.54)			4.36 (2.16)				4.73 (2.55)				4.20 (2.62)			3.60 (2.59)			2.29 (2.43)					1.93 (1.71)
**Dimenhydrinate**																											
	Female				8			1				2				—			—			2					3
	Male				5			—				1				4			—			—					—

^a^Not applicable.

^b^PHQ-9: Patient Health Questionnaire-9.

### Professions Tend to Perform Best in Situation Monitoring and Worst in Mutual Support

Table 1 presents descriptive statistics of the TeamSTEPPS scores, with each simulated profession assessed independently by 2 raters (Rater 1 and Rater 2). A total of 12 raters were involved in the study. The statistics were calculated from 22 sessions of 11 radiological technologists, 30 sessions of 15 medial technologists, 30 sessions of 15 medical doctors, 30 sessions of 15 pharmacists, 30 sessions of 15 airway nurses, and 28 sessions of 14 circulation nurses. To enable comparisons across professions and to compute the group TeamSTEPPS score, participants’ scores were scaled to a range between 0 and 1, since the score ranges varied by topic and profession (Table 1, “Range” rows). This resulted in the Scaled AVG values in Table 1, where higher values indicate better performance.

The average interrater agreement for the TeamSTEPPS scores, as measured by the ICC, was good across the 5 topics and 6 professions (mean 0.73, SD 0.18; range 0.32-0.999), with excellent agreement for the overall score (mean 0.87, SD 0.09; range 0.75-0.99). The lowest ICC was 0.32 for mutual support within the circulation nurse profession, whereas the highest was 0.999 for leadership within the medical technologist profession. Team structure yielded the highest average agreement across professions (mean 0.86, SD 0.13; range 0.64-0.997), while mutual support had the lowest (mean 0.60, SD 0.25; range 0.32-0.94). Among professions, medical technologists had the highest overall agreement (mean 0.98, SD 0.03; range 0.94-0.999), and pharmacists had the lowest (mean 0.59, SD 0.14; range 0.43-0.78). For radiological technologists, team structure showed the highest ICC and mutual support the lowest. For medical technologists, leadership scored highest and mutual support lowest. For medical doctors, communication had the highest agreement and situation monitoring the lowest. For pharmacists, circulation nurses, and airway nurses, team structure showed the highest ICC, while situation monitoring (pharmacists and airway nurses) and mutual support (circulation nurses) were lowest.

The results of the TeamSTEPPS performance comparison among professions across topics were summarized in the Scaled AVG rows of Table 1. Medical doctors demonstrated the highest performance in both team structure and situation monitoring compared to other professions. Pharmacists excelled in communication and leadership, while airway nurses performed best in mutual support. Notably, all professions exhibited their lowest Scaled AVG scores in mutual support, except for airway nurses, who had the lowest in communication. Each profession showed distinct strengths in different topics: radiological technologists and circulation nurses excelled in team structure; medical technologists, medical doctors, and airway nurses in situation monitoring, and pharmacists in communication.

Table 1 presents the group TeamSTEPPS scores for the 6 simulated professions across all 20 sessions and for the subset of 9 sessions in which all 6 student TIs were present. In both analyses, the lowest group Scaled AVG value was observed in mutual support, while the highest was observed in situation monitoring for the 20 sessions and in team structure for the 9 sessions. This indicated that the group performed worst in mutual support and best in situation monitoring across the 20 sessions.

### Scenario Dominated by Communication Between Medical Doctors and Airway Nurse Professions

**Figure 5:**
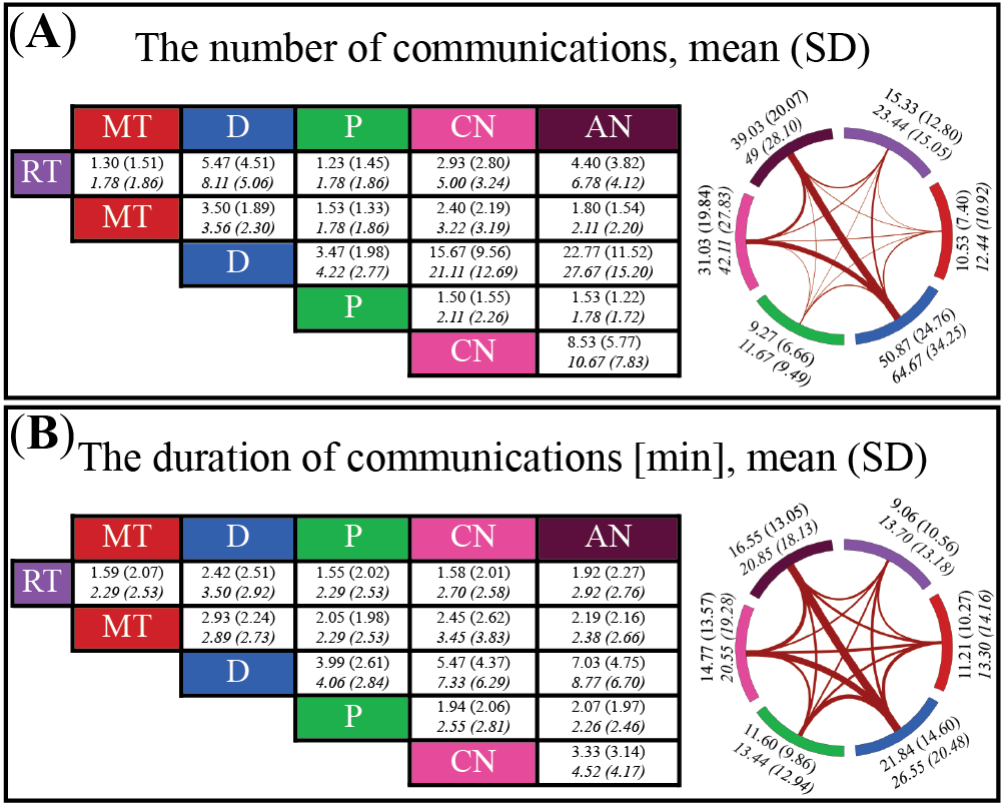
Descriptive statistics of communications between participants. (A) The number of communications among 6 professions (left) and a connectogram (right) where thicker lines indicate more frequent communications. (B) Communication duration in minutes (left) and a connectogram (right) where thicker lines indicate longer durations. Numbers around connectograms indicate totals per profession. Italicized numbers denote complete groups in which all 6 student total interdependence (TI) were present. AN: airway nurse; CN: circulation nurse; D: medical doctor; MT: medical technologist; P: pharmacist; RT: radiological technologist.

We examined the characteristics of communications between professions during the virtually simulated scenario, focusing on frequency and duration. As shown in Figure 5, the medical doctor and airway nurse professions communicated most frequently, with an average of 22.77 (SD 11.52) communications across 30 sessions and 27.67 (SD 15.20) across 9 sessions. Their communications also lasted the longest, averaging 7.03 minutes (SD 4.75 minutes) for the 30 sessions and 8.77 minutes (SD 6.70 minutes) for the 9 sessions. In contrast, communications between the radiological technologist and pharmacist professions were the least frequent (mean 1.23, SD 1.45) and shortest (mean 1.55 minutes, SD 2.02 minutes) across 30 sessions. In the 9 sessions, the shortest communication duration occurred between pharmacist and airway nurse professions (mean 2.26 minutes, SD 2.46 minutes), while the least frequent communications were observed between the following pairs: radiological technologist and medical technologist, radiological technologist and pharmacist, medical technologist and pharmacist, and pharmacist and airway nurse (all mean 1.78, SD 1.86).

### The Alpha-Band Communication TI Deviates More From Baseline Than All Frequency Bands

Table 3 presents descriptive statistics of the communication TI for students across all frequency bands and specifically for the alpha frequency band, in the anterior, central, and posterior brain areas. Data are reported for each profession over the 30 sessions (total sessions) and for the 9 sessions (complete subset), representing the sessions in which the student TI of all 6 simulated professions was fully available.

The absolute values of the alpha-band communication TI of a student were larger than those of all frequency bands in most cases: 78% (14/18) during the 30 sessions and 67% (12/18) during the 9 sessions across the 6 professions and 3 brain areas. In the anterior brain area, 83% (5/6) of the professions showed higher alpha-band values during the 30 sessions, and 67% (4/6) during the 9 sessions. In the central brain area, this trend persisted for 83% of the professions in both sets, whereas in the posterior brain area, it was 67% for the 30 sessions and 50% for the 9 sessions. These results suggest that the alpha-band communication TI of a student deviated more from baseline and conveyed more information about brain-to-brain synchronization than the all frequency-bands communication TI.

We compared the absolute values of the alpha-band communication TI of a student across the 3 brain areas for each profession. In the 30 sessions, the central brain area exhibited larger values than the anterior and posterior areas in 67% (4/6) of professions, while the posterior area showed larger values in 33% (2/6) of professions, and no profession showed larger values in the anterior area. In the 9 sessions, larger values were observed in the anterior, central, and posterior brain areas in 33% (2/6), 17% (1/6), and 50% (3/6) of professions, respectively. The 30-session results suggest that the alpha-band communication TI in the anterior brain area may least represent brain-to-brain synchronization, while the 9-session results suggest a moderate representation in the anterior brain area.

**Table 3:** Descriptive statistics of the communication total interdependence of a student. Please note that, statistics were calculated after removing outliers.

Brain areas			The communication TI^a^ of a student, mean (SD), n										
			RT^b^ (N=22)		MT^c^ (N=30)		D^d^ (N=30)		P^e^ (N=30)		CN^f^ (N=28)		AN^g^ (N=30)
**Anterior**													
	All frequency bands (total sessions)	−0.01 (0.21), 21		0.09 (0.43), 28		0.002 (0.12), 22		−0.002 (0.45), 25		0.01 (0.20), 21		0.003 (0.22), 26	
	All frequency bands (complete subset)^a^	0.04 (0.23), 9		0.04 (0.42), 9		−0.0001 (0.23), 9		0.03 (0.25), 8		−0.08 (0.17), 9		0.09 (0.24), 9	
	Alpha band (total sessions)	0.06 (0.32), 21		0.07 (0.48), 27		0.02 (0.18), 24		−0.08 (0.31), 24		−0.02 (0.24), 22		0.03 (0.24), 26	
	Alpha band (complete subset)^a^	−0.05 (0.11), 8		0.26 (0.74), 9		0.02 (0.17), 9		−0.19 (0.21), 8		0.06 (0.16), 8		0.04 (0.25), 9	
**Central**													
	All frequency bands (total sessions)	0.06 (0.30), 21		−0.04 (0.41), 28		−0.02 (0.18), 24		−0.09 (0.41), 24		0.03 (0.30), 22		−0.04 (0.17), 26	
	All frequency bands (complete subset)^a^	−0.007 (0.21), 9		0.05 (0.44), 9		−0.06 (0.17), 9		−0.13 (0.22), 8		−0.03 (0.27), 9		0.03 (0.15), 9	
	Alpha band (total sessions)	−0.08 (0.29), 21		0.09 (0.51), 28		0.03 (0.19), 24		−0.20 (0.27), 22		−0.004 (0.31), 21		0.10 (0.20), 24	
	Alpha band (complete subset)^a^	−0.15 (0.27), 9		0.13 (0.52), 9		0.03 (0.15), 9		−0.19 (0.25), 8		−0.05 (0.20), 9		0.12 (0.21), 8	
**Posterior**													
	All frequency bands (total sessions)	0.07 (0.26), 20		0.03 (0.41), 28		−0.01 (0.16), 24		−0.01 (0.29), 25		0.0005 (0.24), 20		−0.07 (0.12), 24	
	All frequency bands (complete subset)^a^	0.11 (0.31), 8		−0.22 (0.21), 7		−0.04 (0.18), 9		−0.01 (0.25), 9		−0.11 (0.19), 9		0.01 (0.07), 8	
	Alpha band (total sessions)	−0.002 (0.27), 21		0.07 (0.52), 27		0.05 (0.22), 24		−0.07 (0.23), 22		−0.03 (0.32), 22		0.07 (0.18), 23	
	Alpha band (complete subset)^a^	−0.006 (0.15), 9		−0.06 (0.39), 8		0.07 (0.05), 7		−0.11 (0.19), 8		−0.07 (0.32), 9		0.14 (0.12), 9	

^a^This corresponds to the complete subsets, in which all 6 student TIs were present.

^b^RT: radiological technologist.

^c^MT: medical technologist.

^d^D: medical doctor.

^e^P: pharmacist.

^f^CN: circulation nurse.

^g^AN: airway nurse.

### Group TI Correlates With Group TeamSTEPPS Score

In this section, we present the correlations between TeamSTEPPS and TI after removing outliers using the Minimum Covariance Determinant method and correcting for multiple comparisons with the Benjamini and Hochberg procedure. The TeamSTEPPS scores included 5 topics—team structure, communication, leadership, situation monitoring, and mutual support—along with an overall score. The TI considered here comprised both the student TI and the group TI.

Initially, we examined the correlations between the TeamSTEPPS scores and the student TI. For all 6 simulated professions across both the all frequency and alpha bands, the correlations were weak and not statistically significant (all adjusted *P*≥.05; Figures S2-S7 in Multimedia Appendix 7), except for the medical technologist simulated profession, which showed a significant negative correlation with the team structure topic in the alpha band at the anterior brain area (*r*=–0.76 adjusted *P*<.001). Combining data from all 6 simulated professions also resulted in weak and not statistically significant correlations for both frequency bands (adjusted *P*>.99; Figure S1 in Multimedia Appendix 7).

**Figure 6:**
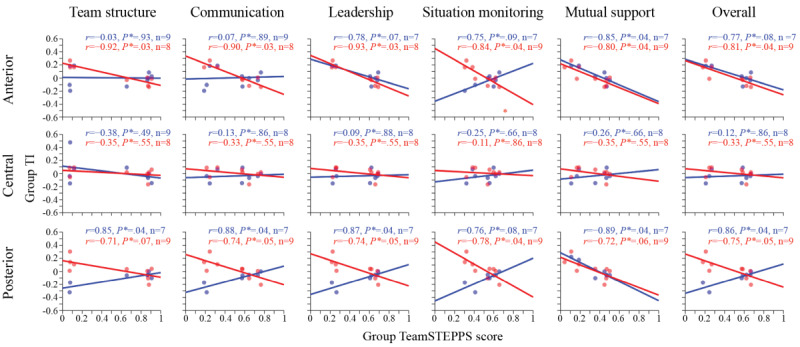
Anterior group total interdependence (TI) in the alpha band negatively correlated with group Team Strategies and Tools to Enhance Performance and Patient Safety (TeamSTEPPS) scores. Scatter plots show relationships between group TI and TeamSTEPPS scores for anterior (top), central (middle), and posterior (bottom) brain areas, across all frequency bands (blue) and only the alpha band (red). Columns represent TeamSTEPPS domains, with the last column showing their sum. Legends display Pearson correlation (*r*), adjusted *P* value (P*), and sample size after excluding outliers (n). Best-fit least-squares lines are shown.

Second, we examined the correlations between the group TeamSTEPPS scores and the group TI, with results presented in Figure 6. This figure shows scatter plots illustrating the associations between the group TeamSTEPPS scores and the group TI for all frequency bands (blue) and the alpha frequency band (red) in the anterior (top row), central (middle), and posterior (bottom row) brain areas. The scatter plots are overlaid with the best-fit lines calculated using the least-squares method (*polyfit* function of MATLAB, The MathWorks, Inc). Out of the 30 sessions, a total of 9 sessions had complete student TI data for all 6 simulated professions, resulting in well-defined group TIs. Note that the number of sessions shown in the scatter plots may be lower than 9 due to the outlier screening procedure.

In the anterior brain area (Figure 6, top row), the alpha frequency band demonstrated strongly negative and statistically significant correlations with all 5 TeamSTEPPS topics, including the overall score: *r*=–0.92, adjusted *P*=.03 for team structure; *r*=–0.90, adjusted *P*=.03 for communication; *r*=–0.93, adjusted *P*=.03 for leadership; *r*=–0.84, adjusted *P*=.04 for situation monitoring; *r*=–0.80, adjusted *P*=.04 for mutual support; and *r*=–0.81, adjusted *P*=.04 for the overall score. In contrast, all frequency bands yielded strongly negative but nonstatistically significant correlations for the other topics, except for mutual support: *r*=–0.03, adjusted *P*=.93 for team structure; *r*=0.07, adjusted *P*=.89 for communication; *r*=–0.78, adjusted *P*=.07 for leadership; *r*=0.75, adjusted *P*=.09 for situation monitoring; *r*=–0.85, adjusted *P*=.04 for mutual support; and *r*=–0.77, adjusted *P*=.08 for the overall score.

In the central brain area (Figure 6, middle row), both all frequency bands and the alpha frequency band exhibited weak, nonstatistically significant correlations for all 5 topics including the overall score, with minimum adjusted *P*=.49 and a maximum absolute *r*=0.38.

In the posterior brain area (Figure 6, bottom row), all frequency bands displayed strongly positive and statistically significant correlations for team structure (*r*=0.85, adjusted *P*=.04), communication (*r*=0.88, adjusted *P*=.04), leadership (*r*=0.87, adjusted *P*=.04), and the overall score (*r*=0.86, adjusted *P*=.04). There were also strongly negative and statistically significant correlations for mutual support (*r*=–0.89, adjusted *P*=.04) and a strongly positive but nonstatistically significant correlation for situation monitoring (*r*=0.76, adjusted *P*=.08). The alpha frequency band in the posterior brain area showed strongly negative and statistically significant correlations for situation monitoring (*r*=–0.78, adjusted *P*=.04), but strongly negative and nonstatistically significant correlations for team structure (*r*=–0.71, adjusted *P*=.07), communication (*r*=–0.74, adjusted *P*=.05), leadership (*r*=–0.74, adjusted *P*=.05), mutual support (*r*=–0.72, adjusted *P*=.06), and the overall score (*r*=–0.75, adjusted *P*=.05).

## Discussion

### Principal Findings

This study explored the correlation between brain-to-brain synchronization, measured by the TI, and team performance, evaluated using the TeamSTEPPS scores, in an online virtual SIMBIE setting where face-to-face communication was absent. At the individual level, no significant correlations were found between participants’ synchronization with their team members (measured by the student TI) and their TeamSTEPPS scores (all adjusted *P*≥.05). These findings are consistent with previous studies [57,58], which similarly reported an absence of brain-to-brain synchronization when face-to-face interactions—crucial mechanisms such as eye contact, important for establishing trust and enhancing synchrony [59]—were missing.

However, at the team level, we identified strongly negative, statistically significant correlations between the group TIs and the group TeamSTEPPS scores, particularly in the anterior brain region (mean −0.87, SD 0.06; range −0.93 to −0.8). These findings are counterintuitive and contrast with our initial hypothesis, as higher team performance is generally associated with greater brain-to-brain synchronization [8]. To better understand this result, we examined the assessment method: team performance was evaluated using the TeamSTEPPS scores, which heavily rely on effective verbal communication [28]. Our findings tentatively suggest that a high degree of brain-to-brain synchronization does not necessarily correspond to improved team performance. Conversely, at the team level, strongly positive, statistically significant correlations were observed between the group TIs based on all frequency bands and the group TeamSTEPPS scores—including the overall score and most topics—particularly in the posterior brain region.

### Comparison With Previous Work

To our knowledge, no previous studies have examined the correlation between EEG-based brain-to-brain synchronization and TeamSTEPPS scores in an online hexad virtual SIMBIE setting without face-to-face communication. This study provides a novel contribution by incorporating a broader set of 6 participants and addressing an important gap in the literature on team-based interactions, using a cost-effective EEG device for analysis.

### Exploring the Discrepancy: Negative Correlation of Anterior Alpha Band With TeamSTEPPS Performance and the Role of Intermittent Synchronization in Hexad Multi-Person Teams

Previous studies generally reported a positive correlation between brain-to-brain synchronization and team performance using basic social interaction tasks, such as Lego assembly, typically conducted in face-to-face dyadic groups and using high-cost methods, including functional near-infrared spectroscopy, near-infrared spectroscopy–based hyperscanning, and resource-intensive EEG studies [8,18,60-63]. In contrast, this study observed strongly negative, statistically significant correlations between team brain-to-brain synchronization—measured by the group TIs based on the anterior alpha band—and team performance assessed using the group TeamSTEPPS scores, including all 5 individual domains and their overall sum. By comparison, group TIs in the posterior frequency bands showed strongly positive, statistically significant correlations with team performance in nearly all individual domains and the overall sum.

We speculate that the discrepancy between the negative correlations observed in this study based on the anterior alpha band and the positive correlations reported in previous studies may result from the complexity of more-than-two-person communication in this study and the nature of the TeamSTEPPS scores, in contrast to previous studies focused on dyadic interactions [61-63]. For instance, compared to face-to-face dyads, groups with more than 2 individuals exhibit distinct patterns in verbal communication and nonverbal behaviors (eg, eye gaze) [64,65], which require intermittent synchronization—the brain’s ability to shift focus among individuals—to maintain effective communication and coordination [66,67]. Intermittent synchronization in groups of more than 2 members was also observed in Stevens et al’s [17,21] studies, which used mannequin-based group simulations with face-to-face interactions. These studies reported that triad groups exhibiting high TeamSTEPPS scores demonstrated elevated Shannon entropy of neurodynamic symbols derived from neural activity at the 10-Hz brain rhythm. Because the 10-Hz rhythm falls within the alpha frequency band and is associated with attention regulation, high Shannon entropy of the corresponding neurodynamic symbols likely reflects the waxing and waning of group members’ attention during the simulation.

In our experimental setting, where 6 participants worked as a team, the brain’s ability to engage in intermittent synchronization was crucial for effective communication and coordination [66,67]. Over synchronization within a team of more than 2 members may be detrimental to team performance, potentially leading to groupthink, a phenomenon in which members prioritize harmony and consensus over critical thinking and individual opinions [67,68].

The TeamSTEPPS scores rely heavily on effective verbal communication to establish a shared mental model of team tasks, strategies, and goals, which is critical for enhancing situational awareness and overall team performance [28,69-71]. TeamSTEPPS promotes an ideal team typology that requires intermittent synchronization—the ability to shift focus and support shared decision-making [72]. However, over synchronization, or an inability to shift focus among more-than-two team members, may disrupt communication and lead to groupthink [67,68]. In such cases, directive leadership and hierarchical structures within health care teams can prioritize consensus at the expense of independent critical thinking, potentially resulting in negative outcomes and impeding shared decision-making [68]. Groupthink, or cohesive typology, relies on a lower cognitive level than a shared mental model or facilitated team structure [72]. This may explain why higher brain-to-brain synchronization, or over-synchronization, as measured by group TIs, does not necessarily translate into optimal team performance as assessed by the TeamSTEPPS scores.

### Anterior Alpha Band Synchronization: A Key Indicator for TeamSTEPPS Performance and Shared Attention in Hexad Teams Without Face-to-Face Interaction

The statistically significant correlations between brain-to-brain synchronization at the team level and TeamSTEPPS performance were primarily observed in the anterior brain areas, particularly in the alpha band. In other words, the anterior electrodes provided the most reliable measurements of TeamSTEPPS performance both overall and across each of the 5 domains, compared to the central and posterior electrodes. These findings align with previous studies that emphasize the critical role of the dorsomedial prefrontal cortex, which is associated with the perception of intention [73,74] and theory of mind, the ability to interpret others’ mental states [75,76], as well as the frontopolar region, a part of the prefrontal cortex, during social interactions [77,78]. However, our results differ from other studies that identified different brain regions, such as the right temporo-parietal junction, as being highly activated during social interaction [57,79]. This discrepancy may arise from variations in the experimental conditions and the nature of tasks used [10].

Unlike the alpha band (8-12 Hz), the delta (1.5-4 Hz [80]), theta (4-8 Hz [81]), and beta (14-30 Hz [82]) bands were not analyzed individually due to limited theoretical support, as their primary functions are less relevant in studies focused on teamwork and attention. Supporting our findings, previous research has identified a relationship between brain-to-brain synchronization in the alpha band and interactional synchrony during social interactions [61,63,83-87]. While some studies suggested that the gamma band (30-80 Hz) may also reflect brain-to-brain synchronization and shared intentions [88], the sampling rate of our EEG device (125 Hz), with a Nyquist frequency of 62.5 Hz limited our analysis to frequencies up to the beta band (14-30 Hz), thus preventing examination of the gamma band. Our emphasis on the alpha band is substantiated by its established roles in attention regulation [44,45,89,90] and cognitive control [46,47]. The alpha band has been extensively studied in contexts such as relaxation [91], inhibitory control [92], emotional processing [93], mental health [94], and sleep [95], making it a versatile marker for various cognitive processes. Our findings further highlight the value of the alpha band as an indicator of fluctuations in attention and focus during team-based activities as measured by TeamSTEPPS.

### Overcoming Subjectivity in TeamSTEPPS Assessment: The Potential of Anterior Alpha Band EEG Synchronization in SIMBIE Scenarios

Figure 5 highlights differences in communication duration among participants, with medical doctors having the longest duration, which may explain their higher TeamSTEPPS scores. In contrast, radiological technologists, with the shortest communication duration, may have lacked closed-loop communication and sufficient identification, impacting critical information sharing as emphasized by the “Introduction, Situation, Background, Assessment, Recommendation” guidelines [96], and resulting in lower TeamSTEPPS scores. Interestingly, despite limited communication duration, the pharmacists achieved a relatively high TeamSTEPPS score. This may be due to the scenario design, where the pharmacist’s role involved gathering medication history from a nonplayer character acting as a family member, thereby boosting TeamSTEPPS scores despite minimal communication with the team. However, no significant correlation was observed between the communication TI of a pair and communication duration, except for a low-value correlation in the central brain area within the alpha frequency band (Figure S1 in Multimedia Appendix 8).

The TeamSTEPPS scores in this study were derived from both verbal communications and nonverbal behaviors. The ICC values for the situation monitoring and mutual support domains were the lowest among the 5 domains (Table 1), likely due to their reliance on nonverbal behaviors and the need for experienced raters to assess them accurately. Examples include monitoring and checking patient’s vital signs before, during, and after intubation, during X-ray procedures, and before intensive care unit transfer, as well as mutual assistance tasks such as raising bed rails and assisting with donning personal protective equipment. These findings indicate that assessing situation monitoring and mutual support with human raters is inherently limited by the subjectivity required to evaluate nonverbal behaviors [97]. However, EEG brain-to-brain synchronization, particularly in the anterior alpha band, offers a promising alternative method to support and enhance the prediction of TeamSTEPPS performance across all domains (Figure 6). The inherent subjectivity of human raters in evaluating situation monitoring and mutual support domains presents a challenge that could be intriguingly addressed by leveraging measures from the anterior alpha band. Previous studies [98-101] have used alpha-band EEG to measure individual situation awareness in real time. In contrast, our findings emphasize the potential of anterior alpha-band synchronization as a group-level metric, offering a fresh perspective on assessing team dynamics and collaborative performance.

### Posterior All-Band Synchronization as a Potential Marker for Certain TeamSTEPPS Domains and Task-Dependent Roles in Hexad Teams Without Face-to-Face Interaction

Our results revealed strong positive and statistically significant correlations between the posterior group TIs based on all frequency bands (1-20 Hz, encompassing partial delta, theta, alpha, and partial beta) and the group TeamSTEPPS scores across most domains (*r*=0.76-0.88). Notably, the mutual support domain deviated from this pattern, showing a strong negative correlation (*r*=−0.89). The positive correlations between team performance and brain-to-brain synchronization across all frequency bands align with previous studies [8,14], which hypothesized that brain-to-brain synchronization is modulated by shared attention among team members. However, our results based on the posterior alpha band, which has a putative role in attention [44,45,89,90], showed the negative correlations that contrasted both with the posterior all frequency band results in this study and with previous studies based on all frequency bands.

Grounded in previous findings, the delta, theta, and beta frequency bands have well-known functional roles beyond attention—for example, levels of consciousness for the delta band [102], cognitive performance for the theta band [103], and motor control and execution for the beta band [104]. Our results tentatively suggest that in the complex interactions among the 6 team members in this study, brain-to-brain synchronization across all frequency bands cannot be explained solely by the shared attention mechanism. Therefore, we recommended that future research investigate brain-to-brain synchronization for each frequency band separately to understand their functional roles during cooperation and social interaction in teams with a larger number of participants, as in this study.

A recent study by Reinero et al [8] explored the relationship between team performance on problem-solving tasks and interbrain synchrony within a group of 4 participants. They found that higher interbrain synchrony, reflecting stronger connections among teammates, led to better performance on economic games and most problem-solving tasks. In contrast, this study found that higher interbrain synchrony correlated with lower TeamSTEPPS performance. Several factors may explain this difference. First, this study was conducted in a virtual environment without face-to-face interaction, which is known to reduce interbrain synchrony [8,57,58]. Second, our tasks required critical thinking and rapid, shared decision-making under time constraints, making effective verbal communication essential. With a larger team of 6—exceeding the arguably optimal team size of 4 [105]—intermittent synchronization, or the brain’s ability to shift focus among teammates, was crucial. Over interbrain synchrony may have disrupted this focus shifting, impeding team performance. In the study by Reinero et al [8], however, the team had fewer time pressures and communicated through private online chat, making intermittent synchronization less critical and allowing high interbrain synchrony to enhance performance. Our findings, together with those of Kikuchi et al [58] and Czeszumski et al [10], suggest that both the context of brain-to-brain synchronization and task characteristics are important factors in the relationship between brain-to-brain synchronization and team performance.

### Strengths and Limitations

Excessive EEG noise during gameplay resulted in group TIs being available for only 45% (9/20) of sessions. Although correlations between group TIs and the TeamSTEPPS scores were statistically significant after correction for multiple comparisons, a minimum of 30 sessions is typically recommended to achieve meaningful correlations [106]. To account for potential data loss of up to 55% under similar conditions, we recommend that researchers plan for at least 67 sessions (100×30/45).

This study focused on healthy participants by excluding individuals with neurological, psychiatric, or substance use disorders, which are known to alter social cognition [107], emotional processing [108], or attentional processing [109]—mechanisms fundamental to brain-to-brain synchronization [110]. Several studies have reported that such disorders can influence brain-to-brain synchronization between individuals during various tasks. For instance, Deng et al [111] conducted an EEG hyperscanning study examining brain-to-brain synchronization during an emotional processing task in 25 parent-adolescent pairs with social anxiety, and found that the adolescents’ level of social anxiety modulated the synchronization. Similarly, Wang et al [112] reported that the severity of autism spectrum disorder in children affected brain-to-brain synchronization during a cooperative task with their parent, although Kruppa et al [113] found no such modulation. Therefore, the applicability of our findings should be interpreted with caution in practical settings where screening for such disorders may not be feasible.

Time accuracy and precision are critical in brain-to-brain synchronization research. While our methodology produced statistically significant correlations and passed sanity checks, there is potential for further refinement. The first improvement is the Bluetooth connection between the EEG data-acquisition device and the PC. A higher baud rate can reduce transmission time but it also increases vulnerability to errors from electromagnetic interference. In this study, we set the baud rate to 115,200 bits per second, which theoretically allows a 24-bit data point to be transferred in 0.2083 milliseconds. However, factors such as distance, interference, Bluetooth overhead, and system delays in Windows 10 can extend transmission times to 0.6-10 milliseconds [114]. Despite these latency variations, BrainFlow software (version 4.9.0 [41]) maintained the correct order of data points. The second improvement involves better alignment of the 6 recorded screen footages. In this study, we manually aligned the footages using commercial software, which introduced the risk of human error, potentially causing partial loss of verbal communication data and related EEG information. We recommend real-time audio logging for both senders and receivers during virtual simulations to accurately capture team audio profiles. The third improvement focuses on enhancing the accuracy of the local Unix time. We used the C# DateTime function, which can differ from real time by approximately 10 milliseconds [115]. Implementing dedicated external hardware to synchronize time across EEG devices and team PCs would enhance both accuracy and precision.

In this study, we used a fully manual method to reject artifact portions of EEG signals. This approach poses several challenges: (1) potential data bias, (2) time consumption for large-scale studies, (3) reliance on EEG specialists, and (4) limited practicality for real-world applications. Artifact rejection remains a significant bottleneck in EEG data preprocessing, as unaddressed noise and artifacts can impair subsequent analysis. Despite its importance, there has been no consensus within the EEG community on effective artifact management. Recently, various tools and techniques for semiautomatic artifact rejection during offline analysis have emerged, such as the Clean Rawdata EEGLAB plug-in [116,117], Autoreject [118], FASTER [119], the Riemannian Potato technique [120], and the Robust Regression technique [121]. However, these methods often require manual parameter tuning, which can vary depending on the dataset.

Simulator sickness is a common issue in virtual simulations [122]. In this study, of the 90 participants, 13 (14%) took dimenhydrinate before the experiments to prevent nausea, vomiting, and dizziness associated with simulator sickness [123]. While dimenhydrinate can cause side effects such as drowsiness and hyperactivity, which may potentially impact EEG signals [29], Hu [124] reported no changes in peak frequency or the percentage distribution of the alpha band. Therefore, we speculate that our primary finding—the correlation between the alpha group TIs and the group TeamSTEPPS scores—was likely unaffected by dimenhydrinate, though it may have influenced results based on all frequency bands.

Our analysis approach evaluates the normalized TI immediately after each communication, rather than waiting until the end of the entire session. This allows for near real-time computation, making the method practical for real-world applications. This approach requires extensive computation, involving 1000 permutations to construct the TI empirical distribution for normalization. A small percentage of these distributions (175/4224, 4.14%) did not pass the Kolmogorov-Smirnov normality test, suggesting that a higher number of permutations may be needed, which would further increase computational demands.

This study was conducted in a single-room lab without electromagnetic shielding, where all 6 participants shared the same space due to facility limitations. This setup introduced unavoidable environmental factors, such as shared sounds, visuals, and potential interference from external electromagnetic sources, which may have affected EEG signals [125-127]. To reduce spurious brain-to-brain synchronization from these external influences, we recommend that future studies place each participant in a separate, electromagnetically shielded room.

### Future Research

Building on this study and considering the non-face-to-face context of the online hexadic virtual SIMBIE, we recommended using alpha-band activity in the anterior brain regions to evaluate TeamSTEPPS scores. This approach showed significant associations with all 5 TeamSTEPPS domains and provides a valuable framework for understanding shared attention and the development of shared mental models. Additionally, focusing on the anterior brain region is practical because affordable EEG devices with anterior electrodes are readily available, making this method cost-effective and widely accessible for broader applications.

Can we enhance the correlation between EEG brain-to-brain synchronization and TeamSTEPPS scores in virtual simulations or real-life practice to address the challenges of subjective TeamSTEPPS evaluations by raters? Our current methodology relies on verbal communication within a team of at least 2 collaborators to compute TIs. However, the TeamSTEPPS scores also encompass nonverbal components, such as early stages of situation monitoring (eg, visual attention) and mutual support in actions that are not captured by our TI calculations. Future studies could improve TIs by incorporating screen footage segments representing situation monitoring and mutual support, providing a more comprehensive analysis of both verbal and nonverbal components of TeamSTEPPS scores. In real-life clinical practice, it may be feasible to estimate the TeamSTEPPS scores in real-time using affordable, compact EEG devices positioned near the anterior brain region, enabling broader generalizability and practical application across various settings.

### Conclusions

This study found no correlations between individual brain-to-brain synchronization, as measured by the student TI, and individual TeamSTEPPS performance during online virtual SIMBIE sessions without face-to-face communication. However, at the group level, strongly negative, statistically significant correlations were primarily observed in the alpha band in the anterior brain region between the group TI and the group TeamSTEPPS scores among teams of 6 multiprofessional students. These findings highlight the potential of EEG-based brain-to-brain synchronization analysis as an emerging tool for a more objective measure of TeamSTEPPS dynamics than subjective human evaluations, offering a novel approach to support TeamSTEPPS assessments with trend-based, quantitative, real-time feedback—ultimately enhancing team performance and patient safety.

## Appendix

Multimedia Appendix 1 Total Interdependence.

Multimedia Appendix 2 Virtual Simulation Content Overview.

Multimedia Appendix 3 Device specifications.

Multimedia Appendix 4 Experimental settings.

Multimedia Appendix 5 Sanity Checks of the EEG Acquisition System.

Multimedia Appendix 6 EEG Preprocessing.

Multimedia Appendix 7 Correlation between Student TI and the TeamSTEPPS Scores.

Multimedia Appendix 8 Correlation between the Communication TI of a Pair and Characteristics of Communications.

## Abbreviations

EEG: Electroencephalogram
ICC: Intraclass correlation coefficient
PC: Personal computer
PHQ-9: Patient Health Questionnaire-9
SIMBIE: Simulation-based interprofessional education
TeamSTEPPS: Team Strategies and Tools to Enhance Performance and Patient Safety
TI: Total interdependence
VR: Virtual reality
