# Supplementary material for: Correlation Between Electroencephalogram Brain-to-Brain Synchronization and Team Strategies and Tools to Enhance Performance and Patient Safety Scores During Online Hexad Virtual Simulation-Based Interprofessional Education: Cross-Sectional Correlational Study
Source: JMIR Med Educ. 2025 Oct 20;11:e69725. doi: 10.2196/69725 (PMC12583944; doi:10.2196/69725)
Supplement: Multimedia Appendix 1 [file mededu_v11i1e69725_app1.docx]

## Multimedia Appendix 1

Total Interdependence.

### Mathematical formulation of total interdependence.

Mathematical formalism of total interdependence (TI) was laid by Gel’fand and Yaglom [1] and operationalized in the frequency domain of the time series by Geweke [2]. TI can be decomposed into 3 components: 2 directional causal influences due to their interaction patterns, and the instantaneous causality due to factors, e.g., a common driving input [3]: this justifies the word total interdependence.

Given 2 continuous time series $x\left( t \right)$ and $y\left( t \right)$, TI evaluated at a rescaled frequency $\lambda$ is defined by

|  | $TI_{x,y}\left( \lambda\right) = -\frac{1}{2\pi}\int_{-\pi}^{\pi} ln\left[ 1-C_{x,y}^{2}\left( \lambda\right) \right]d\lambda\text{,}$ | **(1)** |
| --- | --- | --- |

where $C_{x,y}$ was the coherence between 2 time series at frequency $f=\lambda/{2\pi}$. Practically, the continuous time series were sampled at some sampling frequency $f_{s}$, i.e., 125 Hz with OpenBCI used in our study. Equation 1 was casted to its numerical version as

|  | $TI_{x,y}\left( f_{i} \right) = -\frac{2}{f_{s}}\sum_{i=1}^{N-1} ln\left[ 1-C_{x,y}^{2}\left( f_{i} \right) \right]\Delta f \text{,}$ | **(2)** |
| --- | --- | --- |

where $f_{i}:=i\Delta f$, $\Delta f$ was the frequency resolution defined as ${f_{s}}/{2\left( N-1 \right)}$, and $N$ is the number of desired frequency points between 0 and ${f_{s}}/2$ (including both ends); that is, $2\left( N-1 \right)$ samples were recorded. The magnitude-squared coherence $C_{x,y}^{2}$ (Equation 2) was computed from the cross-spectral density $S_{x,y}$ and the autospectral densities $S_{x,x}$ and $S_{y,y}$ by

|  | $S_{x,x}\left( f_{i} \right) = \frac{1}{n}\sum_{j=1}^{n} X\left( t_{j}, f_{i} \right)\cdot X^{*}\left( t_{j},f_{i} \right) \text{,}$ | **(3)** |
| --- | --- | --- |
|  | $S_{y,y}\left( f_{i} \right) = \frac{1}{n}\sum_{j=1}^{n} Y\left( t_{j}, f_{i} \right)\cdot Y^{*}\left( t_{j},f_{i} \right) \text{,}$ | **(4)** |
|  | $S_{x,y}\left( f_{i} \right) = \frac{1}{n}\sum_{j=1}^{n} X\left( t_{j}, f_{i} \right)\cdot Y^{*}\left( t_{j},f_{i} \right) \text{,}$ | **(5)** |
|  | $C_{x,y}^{2}\left( f_{i} \right) = \frac{\left\vert S_{x,y}\left( f_{i} \right) \right\vert^{2}}{S_{x,x}\left( f_{i} \right)S_{y,y}\left( f_{i} \right)} \text{,}$ | **(6)** |

where $t_{j}$ referred to the $j^{th}$ epochs and $n$ to the number of epochs. $X\left( t_{j},f_{i} \right)$ and $Y\left( t_{j},f_{i} \right)$ were the Fourier coefficients of the EEG time series of the $j^{th}$ epoch evaluated at $f_{i}$ Hz.

### Dependence of total interdependence on the range of the duration.

The spectral density of epoched EEG signals was an average of the spectral density of each epoch, see Equations 3-5. The principle of the law of large number [4] implies that the spectral density varies as a function of the number of epochs visually demonstrated in Figure S1. Figure S1 showed examples of the empirical distribution of TI corresponding to 2-epoch (red) and 10-epoch (blue) EEG signals; the data preparation section in the main text provided details of construction of the empirical distributions. The figure clearly showed the influence of the principle of the law of large number on the spectral density; that is, both empirical distributions had different mean and standard deviation. This motivated the study to normalize TI before conducting further analysis.


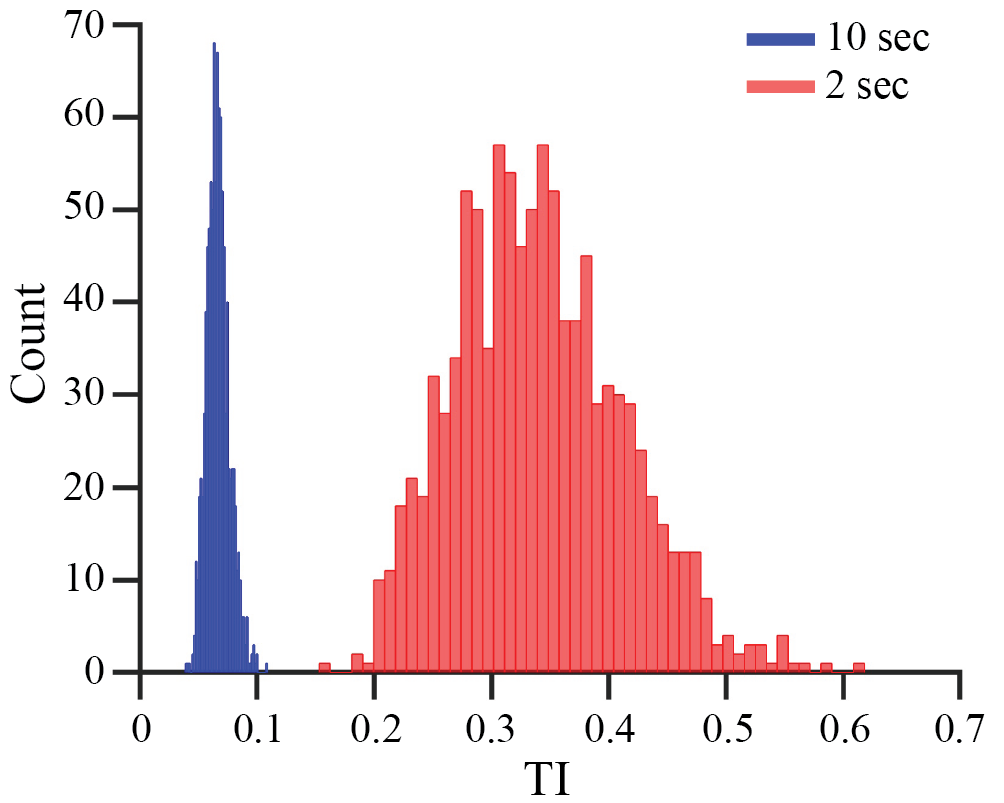


**Figure S1. Total interdependence of signals with different durations.** TI empirical distributions of 2-sec and 10-sec signals are plotted in red and blue, respectively. Both empirical distributions clearly have different mean and standard deviation. **Abbreviations**: TI, Total interdependence.

### Computation of normalized total interdependence.

Out of 1256 communications observed across 30 sessions, a total of 896 valid communications were identified as suitable for TI analysis (mean per session: 29.87, SD: 22.75; range: 2–90). We focused on computing TI for one valid communication, with TI for others calculated analogously. Assume there were *M* participants joining the valid communication (mean 2.29, SD 0.75; range 2-6). Segments of the *M* clean EEG signals corresponding to the valid communication were selected by matching the local Unix timestamps of the clean EEG signals to the refined Unix times of the communication, yielding a total of 2050 EEG segments from the 896 valid communications. We extended any segments shorter than 1 sec to 1 sec in length, resulting in 25 such instances. The *M* segments were then divided into non-overlapping 1-sec epochs. We rejected any epochs if their magnitude in at least one of the 3 EEG averages exceeded 6 times the corresponding golden SDs, defined in Multimedia Appendix 1, resulting in a rejection rate of 0% to 96.30% (mean 9.52%, SD 16.37%; n=2050).

For each pair of *M* participants, we identified common clean epochs that were free from artifacts in both participants; in total, we had 1573 pairs yielding an average of 83.54% (SD 20.10%) of commonly clean epochs during the 896 valid communications. Each selected 1-sec epoch underwent a Hanning windowing and zero-padding to 4 seconds [5] before applying the Fourier transform, which had a frequency resolution of 0.25 Hz (the number of frequency points *N*, 251; see Equation 2 in Multimedia Appendix 2; [6]). We repeated these steps across all commonly clean epochs to calculate *a communication TI of a pair* using Equations 2-6 in Multimedia Appendix 2.

Because the communication TI of a pair depended on the duration of the communication due to the law of large numbers (see Figure S1 in Multimedia Appendix 2; [4]), we normalized the communication TI of a pair as follows. Initially, we randomly selected 1-sec epochs from the clean EEG signals of the pair, matching the number of commonly clean epochs, and repeated this process 1000 times [7]. These randomly selected epochs maintained magnitudes within 6 times the corresponding golden SDs, defined in Multimedia Appendix 1, and did not need to occur simultaneously for both participants. Next, we applied the same computation steps used for the communication TI of the original pair to each of the 1000 randomly selected epochs. This resulted in an empirical distribution of communication TIs for the pair, with 95.86% (4049/4224) of these distributions conforming to a normal distribution, as confirmed by the Kolmogorov–Smirnov normality test at .05 significance level after Bonferroni correction (see Figure S2 in Multimedia Appendix 2). We then normalized the communication TI of the pair using the formula (*TI* – *μ*)/*σ*, where *TI* is the original communication TI, and *μ* and *σ* are the mean and non-biased standard deviation of the empirical distribution, respectively. Independence between the normalized communication TI of the pair and the duration of communications was supported by additional correlation study (see Figure S1 in Multimedia Appendix 6). For the sake of brevity, we omitted the “normalized” term and simply used the communication TI of the pair to represent its normalized version.

### Normality of empirical distributions of total interdependence.

Figure S2 showed empirical distributions with 2 extreme *P* values in the study; the *P* values were obtained from the Kolmogorov–Smirnov normality test (*kstest* function of MATLAB). Panel A of Figure S2 showed the histogram (left column) and the quantile-quantile plot (right column) of the empirical distribution with the lowest *P* value in the study; the empirical distribution was rejected by the normality test (*P*<.001). In contrast, Panel B of Figure S2 showed the plots for the empirical distribution with the highest *P* value in the study; the null hypothesis of the normality test was accepted by the normality test (*P*=.94).


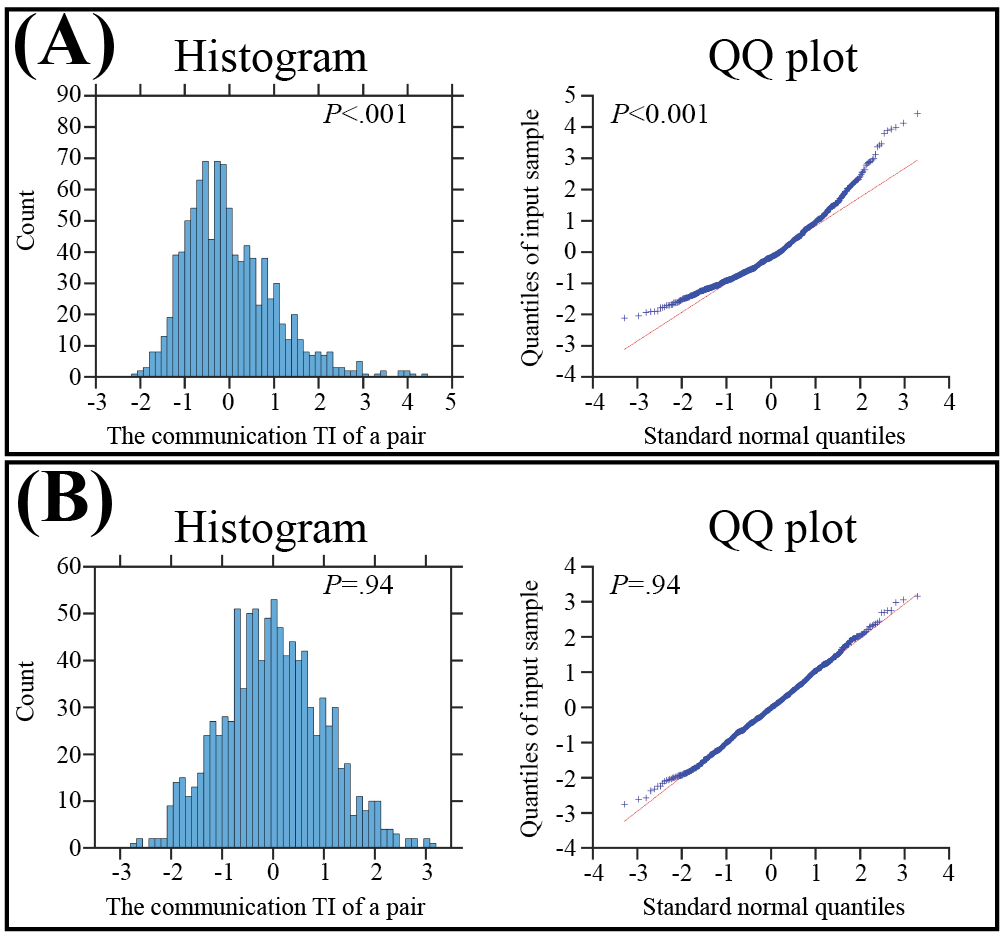


**Figure** **S2. Examples of two extreme empirical distributions.** Panel A shows an empirical distribution that has lowest *P* value among all tested empirical distributions, while Panel B is for highest *P* value. The left column in both panels shows the histogram of the empirical distributions whereas the quantile-quantile plots corresponding to the normal distribution are demonstrated in the right column.

## References

1. Gel’fand IM, Yaglom AM. Calculation of the amount of information about a random function contained in another such function: American Mathematical Society Translations: Series 2; 1959.

2. Geweke J. Measurement of linear dependence and feedback between multiple time series. J Am Stat Assoc. 1982;77(378):304-13. doi: 10.1080/01621459.1982.10477803.

3. Bastos AM, Schoffelen J-M. A tutorial review of functional connectivity analysis methods and their interpretational pitfalls. Front Syst Neurosci. 2016 Jan;9. doi: 10.3389/fnsys.2015.00175.

4. Lamperti JW. Probability: A survey of the mathematical theory. 2nd ed: John Wiley & Sons, Inc.; 1996. ISBN: 0471154075.

5. Dikker S, Wan L, Davidesco I, Kaggen L, Oostrik M, McClintock J, et al. Brain-to-brain synchrony tracks real-world dynamic group interactions in the classroom. Curr Biol. 2017 May;27(9):1375-80. doi: 10.1016/j.cub.2017.04.002.

6. Lalor EC, Kelly SP, Finucane C, Burke R, Smith R, Reilly RB, et al. Steady-state vep-based brain-computer interface control in an immersive 3d gaming environment. EURASIP J Adv Signal Process. 2005 Nov;2005(19):706906. doi: 10.1155/ASP.2005.3156.

7. Cohen MX. Analyzing neural time series data: Theory and practice: The MIT press; 2014. ISBN: 9780262019873.
