# Supplementary material for: Correlation Between Electroencephalogram Brain-to-Brain Synchronization and Team Strategies and Tools to Enhance Performance and Patient Safety Scores During Online Hexad Virtual Simulation-Based Interprofessional Education: Cross-Sectional Correlational Study
Source: JMIR Med Educ. 2025 Oct 20;11:e69725. doi: 10.2196/69725 (PMC12583944; doi:10.2196/69725)
Supplement: Multimedia Appendix 2 [file mededu_v11i1e69725_app2.docx]

## Multimedia Appendix 2

Virtual Simulation Content Overview

The simulation was designed to progressively increase in both complexity and stress, replicating high-acuity emergency scenarios that reflect real-world challenges encountered during the COVID-19 pandemic. It incorporated both technical and non-technical learning objectives within the context of a multidisciplinary emergency department encounter. Six participants, each assuming a distinct role aligned with a fully qualified, licensed healthcare profession, worked collaboratively as a team. The scenario required the team to diagnose and manage a complex case involving a 70-year-old male patient with COVID-19, chronic obstructive pulmonary disease, hypertension, diabetes mellitus, and a documented allergy to ceftriaxone. The inclusion of a distressed spouse in the scenario introduced an emotionally charged element. Therefore, participants would learn both clinical competence and interpersonal communication skills from the scenario.

Technical training encompassed time-sensitive clinical challenges such as intubation, ventilator management, laryngeal mask airway insertion, cricothyroidotomy, management of hyperkalemic crisis, use of personal protective equipment, and coordination of portable chest imaging (see Figures S1C–F for examples). Clinical reasoning was directed toward formulating an accurate diagnosis and implementing a safe treatment plan under pressure, closely mirroring the demands of real-world emergency care environments and emphasizing decision-making under multiple stressors (see Figure S1). Non-technical training focused explicitly on the 5 domains of TeamSTEPPS: (1) team structure; (2) communication; (3) leadership; (4) situation monitoring; and (5) mutual support. Instructional strategies such as STEP, ISBAR, closed-loop communication, and real-time psychosocial peer support [1]. In addition, participants also developed non-technical skills related to managing interactions with distressed family members.

Communication between team members was facilitated through the use of microphones and in-ear speakers, enhancing the fidelity of interprofessional interaction during the simulation. Participants could initiate either an individual call to communicate with a selected team member or a group call to reach the entire team (see Figure S1A with legends “Individual Call” and “Group Call” respectively). However, similar to real-world settings, participants did not need to press a call to communicate when they were in the same virtually simulated room (see Figure S1D). Team members were represented as avatars within the virtual environment and controlled them through the environment from the first-person viewpoint (see Figure S1E for an example). Communication with non-player characters was conducted through a branching dialogue system presented primarily in Thai (see Figure S1B). Non-player characters responded vocally in Thai according to participants’ dialogue selections, contributing to a more immersive and realistic simulation experience (see Figure S1B).


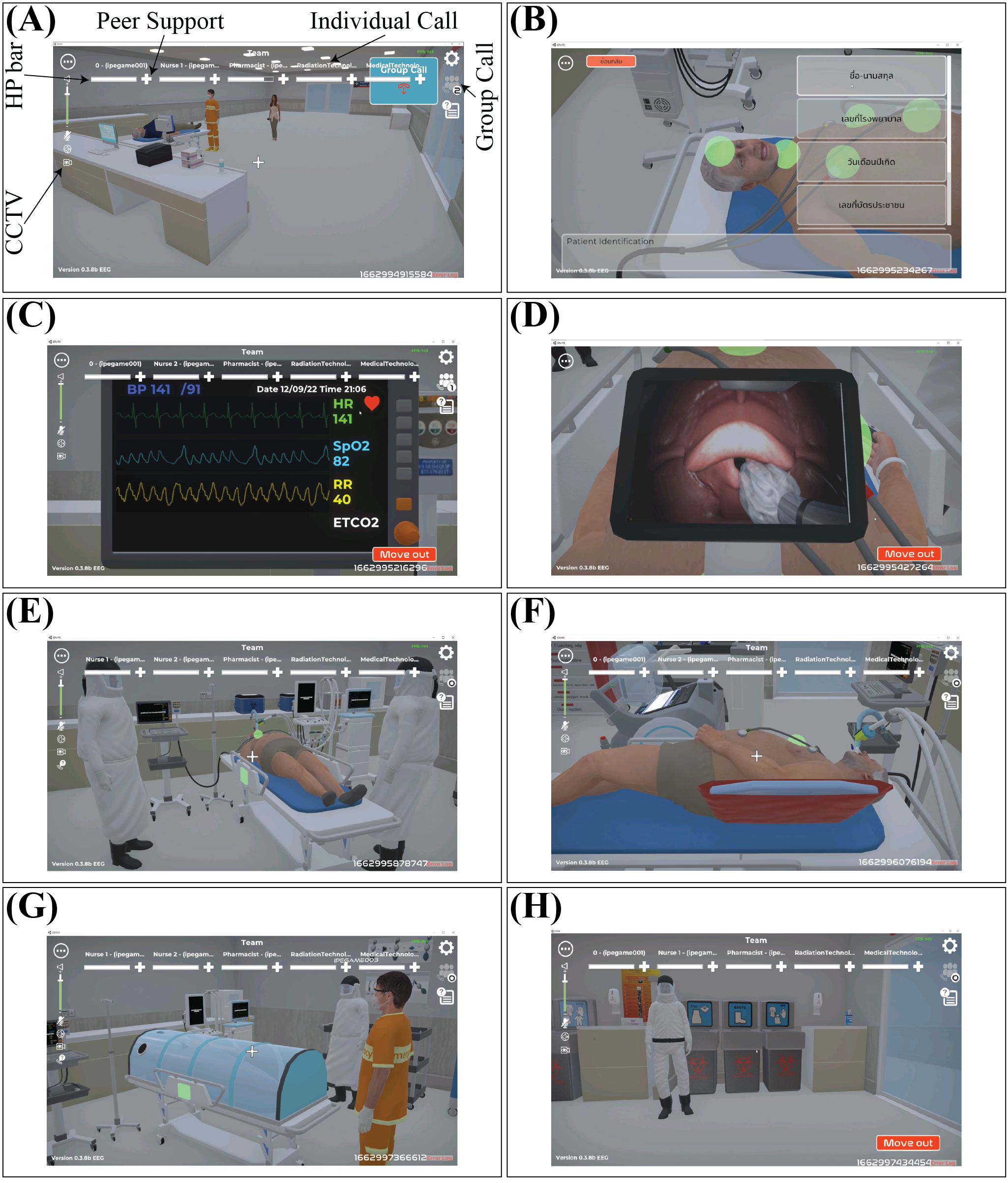


**Figure S1. Content overview of virtually simulated scenario without the VR setting.** Panel A shows the arrival of the patient with his wife. Overview of the main interface, where users control avatars with options for peer support, individual and group calls, and health point monitoring (HP bar). The group call button is often used to initiate team briefings and leadership communication in alignment with TeamSTEPPS principles. In Panel B, non-player characters respond vocally in Thai according to participants’ dialogue selections, contributing to a more immersive and realistic simulation experience. Avatars identify patients via a scripted interaction menu, selecting the correct patient identification wristband and verifying patient information. Panel C shows that avatars can view real-time vital signs on monitors. This scenario depicts a respiratory failure crisis with a high respiratory rate, hypoxia (SpO₂ drop), and early signs of shock (increased HR). Panel D shows “Can’t intubate, can’t ventilate” crisis situation. Failed intubation, despite visible vocal cords, prompts an urgent decision to insert an alternative airway device (e.g., LMA). This is a critical turning point requiring rapid escalation and coordinated teamwork. Panel E shows that team members are represented as avatars within the virtual environment and control them through the environment from the first-person viewpoint. Additionally, Panel E further shows that after successful airway management, interprofessional team members (medical doctor and nurses) share mental models, clarify next steps, and confirm safety—facilitated by direct voice communication when avatars are in proximity (realistic spatial audio). Panel F shows that the team discusses X-ray preparation, ensuring patient safety (e.g., stable oxygenation, secure lines and endotracheal tube, correct patient identification, bed rails up). Collaboration between radiological technologist, medical doctor, and nurses ensures appropriate imaging and diagnosis. Panel G shows that the team prepares for patient transfer, coordinating roles and equipment handover. Communication follows the ISBAR format to ensure safety during the transition. Panel H demonstrates that individual avatars complete PPE doffing according to proper safety protocols. This stage typically involves minimal communication among team members.

The simulation employed an interactive, game-based interface designed to strengthen TeamSTEPPS competencies, which were the core learning objectives. Each participant was assigned a symbolic “health point” (HP) bar (Figure S1A) to represent emotional resilience in high-pressure situations. The HP bar progressively decreased during critical clinical moments or procedural delays—examples include “can’t intubate, can’t ventilate” crises, video laryngoscope battery depletion, or sudden onset of hypotension. A “Peer Support” feature (Figure S1A, indicated by the “plus” button) enabled participants to replenish a teammate’s HP up to five times, serving as a metaphor for mutual support and collective coping. This dual-function design positioned the HP bar as both a real-time stress load indicator and a gamified positive reinforcement tool for behaviors consistent with TeamSTEPPS strategies. These features were intentionally designed to enhance psychological fidelity and simulate cognitive load realism by integrating clinical complexity with emotional dynamics. Additionally, participants could monitor activities occurring in other virtually simulated rooms using the “CCTV” button (see Figure S1A).

Figure S2 illustrated the first-person viewpoint of the medical student within the simulation using the VR setting. The key distinction between the VR and non-VR versions of the simulation lies in the level of immersion. The non-VR simulation employed standard computer input devices (e.g., monitor, keyboard, and mouse), whereas the VR simulation utilized headsets and handheld controllers (see Figure S1D in Multimedia Appendix 3) to enhance emotional engagement and the sense of presence. The VR setting enabled participants to physically move and interact within the virtual environment, creating a fully immersive and realistic experience. This heightened sense of presence made participants feel as though they were truly “inside” the scenario.

**
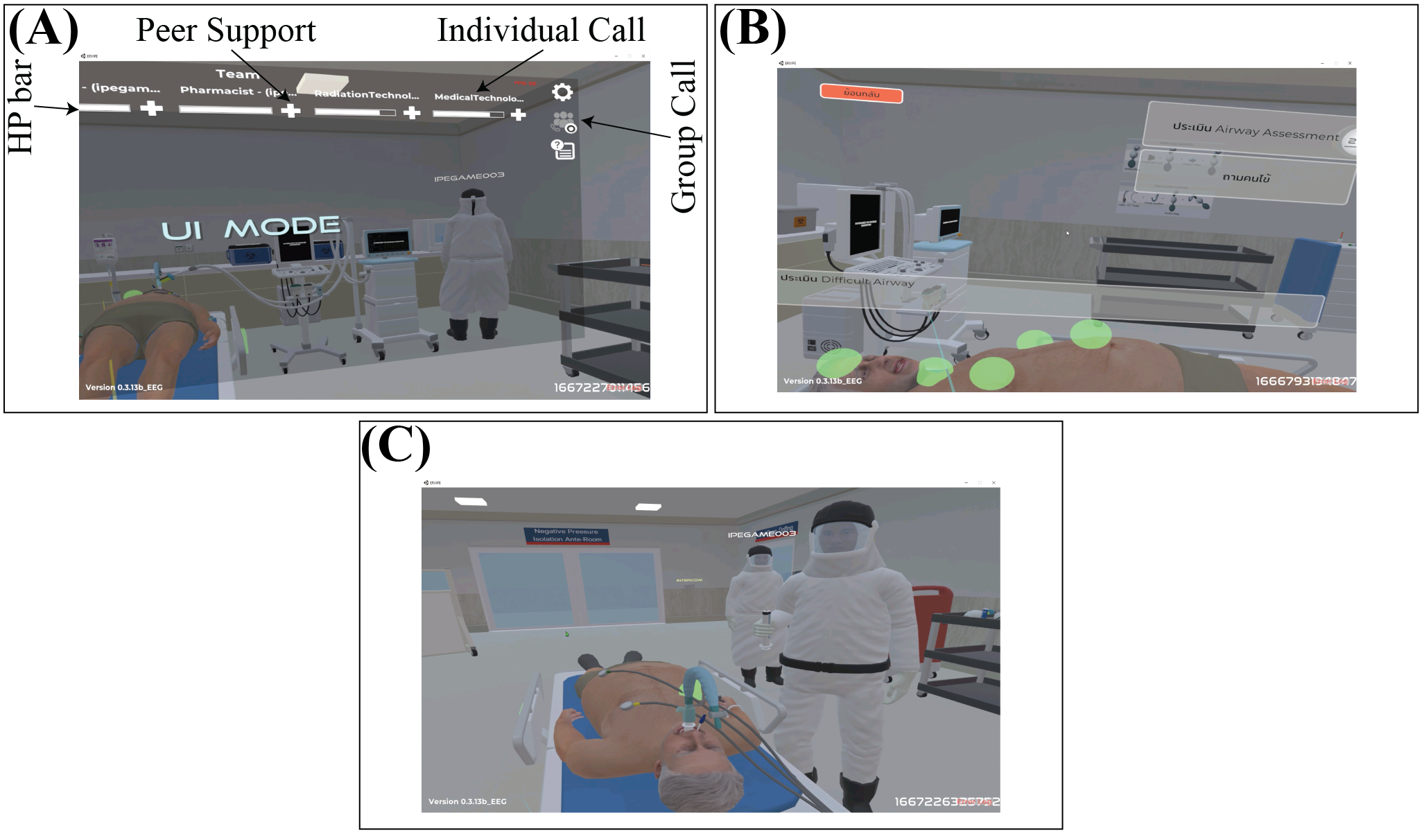
**

**Figure S2. Content overview of virtually simulated scenario with the VR setting.** Panels A and B present the first-person viewpoint of the medical student within scenes similar to those depicted in Figures S1E and S1B, respectively, but rendered in the VR setting. Panel C shows the nurse avatars from the first-person viewpoint of the medical doctor in the VR setting similar to those depicted in Figurer S1E.

## References

1. Buljac-Samardzic M, Doekhie KD, van Wijngaarden JDH. Interventions to improve team effectiveness within health care: a systematic review of the past decade. Hum Resour Health. 2020 Jan 8;18(1):2. PMID: 31915007. doi: 10.1186/s12960-019-0411-3.
