# Supplementary material for: Correlation Between Electroencephalogram Brain-to-Brain Synchronization and Team Strategies and Tools to Enhance Performance and Patient Safety Scores During Online Hexad Virtual Simulation-Based Interprofessional Education: Cross-Sectional Correlational Study
Source: JMIR Med Educ. 2025 Oct 20;11:e69725. doi: 10.2196/69725 (PMC12583944; doi:10.2196/69725)
Supplement: Multimedia Appendix 3 [file mededu_v11i1e69725_app3.docx]

## Multimedia Appendix 3

Device Specifications.


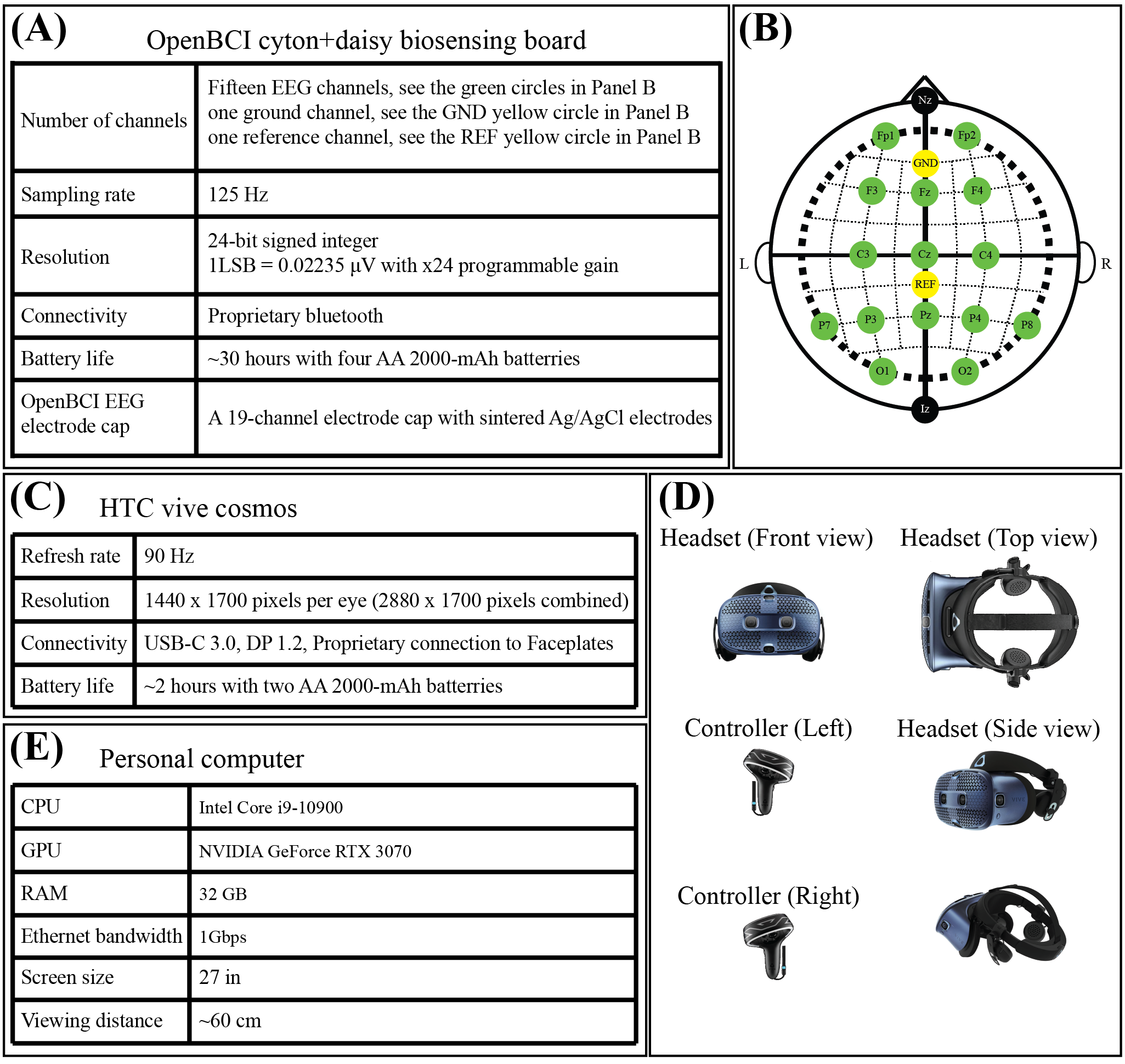


**Figure S1. Device specification of OpenBCI cyton+daisy biosensing board, HTC VIVE Cosmos, and personal computer.** Panel A shows technical details of the OpenBCI acquisition system. Panel B shows the location of the electrodes (green), the reference (REF; yellow), and the ground (GND; yellow) according to the 10-20 system. Panel C shows technical details of the VR headset that consists of devices depicted in Panel D. Panel E demonstrates specifications of the personal computer used in the virtual simulation. **Abbreviations**: C, Central; Cz, Central midline; EEG, Electroencephalogram; F, Frontal; Fp, Frontopolar; GND, Ground; Iz, Inion; LSB, Least significant bit; Nz, Nasion; O, Occipital; P, Parietal; Pz, Parietal midline; REF, Reference.
