# Supplementary material for: Correlation Between Electroencephalogram Brain-to-Brain Synchronization and Team Strategies and Tools to Enhance Performance and Patient Safety Scores During Online Hexad Virtual Simulation-Based Interprofessional Education: Cross-Sectional Correlational Study
Source: JMIR Med Educ. 2025 Oct 20;11:e69725. doi: 10.2196/69725 (PMC12583944; doi:10.2196/69725)
Supplement: Multimedia Appendix 4 [file mededu_v11i1e69725_app4.docx]

## Multimedia Appendix 4

Experimental Settings

### Device setups and their networking.


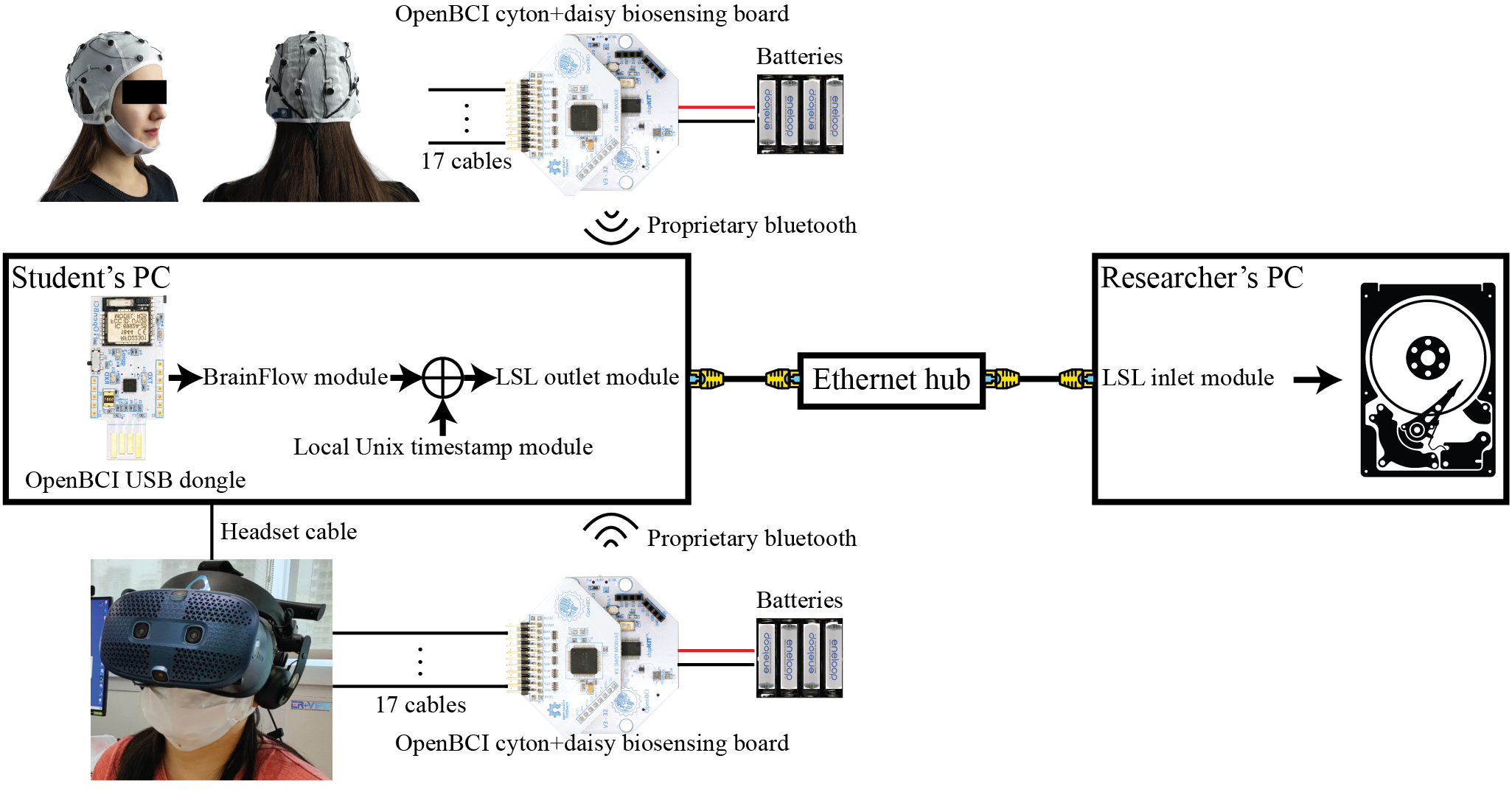


**Figure S1. Device connections.** This figure illustrates the EEG acquisition system with the VR headset in use. In one experimental condition, the participant wears the OpenBCI EEG electrode cap alone, and in another, alongside the VR headset. The electrode cap is connected to the OpenBCI cyton+daisy biosensing board, which samples brain activity and wirelessly transmits the data to the BrainFlow module on the participant’s PC via the OpenBCI USB dongle. The EEG signals are timestamped using the Local Unix timestamp module and subsequently transmitted to the researcher’s PC for storage via the LSL outlet and inlet modules. The de-identified individual is reproduced from https://shop.openbci.com/products/openbci-eeg-electrocap. **Abbreviations**: LSL, Lab streaming layer.

### Laboratory setting.

Six participants including one researcher were in a 7x7m reasonably quiet room, of which temperature was controlled at 23 °C. The space between the adjacent participants was approximately 2 meters, which were distance enough in the limited space mode of the VR headset (see Figure S1D in Multimedia Appendix 3; [1]). Each participant and the researcher had their own personal computer (PC) for independently conducting the experiment and recording data, respectively.

For the sessions without the VR headset, we arranged pieces of equipment by the following. A 27-inch monitor located approximately 60 cm in front of each participant. Brightness of the monitors was adjusted by the participants themselves for their optimal comfortability. The participants communicated to each other in the virtually simulated scenario via an in-ear speaker and a table-standing microphone; loudness of the in-ear speakers was adjusted according to their preference. For the sessions with the VR headset, the participants were aided by trained research assistants (RAs) to install the VR headset together with OpenBCI EEG Electrode Cap (see Figure S1D in Multimedia Appendix 3 and Figure S1 in Multimedia Appendix 4) and support them during the sessions. Furthermore, RAs helped the participants to setup the VR headsets to reduce their simulator sickness [2] and improve immersive experiences of the participants: RAs assisted the participants to perform the Room setup of the VR headset with the Limited space selection [3] and adjust the interpupillary distance [4] before each session began.

The 7 PCs, running on Windows 10 operating system, were composed of Intel Core i9-10900, 32GB DDR4 main memory, and NVIDIA GeForce RTX 3070 as a GPU. They were connected to each other with CAT6 ethernet cable through a gigabit ethernet hub. The ping command between the pairwise PCs in the star network topology informed the pairwise network latency below 1 millisecond. The simulated-scenario server and the EEG data store server executed on the researcher’s PC: The simulated-scenario server was responsible for coordinating participants' virtual simulation games, while the EEG data store server was for real-time monitoring and recording participants' EEG signals. The specifications of PCs and LAN network connectivity ensured smooth experiences of participants during the virtually simulated scenario.

## References

1. HTC Corporation. What is the recommended space for the play area? 2024; <https://www.vive.com/ca/support/cosmos/category_howto/what-is-the-recommended-space-for-play-area.html> [accessed January 8, 2024].

2. Kim HK, Park J, Choi Y, Choe M. Virtual reality sickness questionnaire (vrsq): Motion sickness measurement index in a virtual reality environment. Appl Ergon. 2018 May;69:66-73. doi: 10.1016/j.apergo.2017.12.016.

3. HTC Corporation. Setting up the play area. 2024; <https://www.vive.com/hk/support/cosmos/category_howto/setting-up-the-play-area.html> [accessed January 8, 2024].

4. HTC Corporation. Adjusting the ipd on the headset. 2024; <https://www.vive.com/us/support/cosmos/category_howto/adjusting-the-ipd-on-the-headset.html> [accessed January 8, 2024].
