# Supplementary material for: Correlation Between Electroencephalogram Brain-to-Brain Synchronization and Team Strategies and Tools to Enhance Performance and Patient Safety Scores During Online Hexad Virtual Simulation-Based Interprofessional Education: Cross-Sectional Correlational Study
Source: JMIR Med Educ. 2025 Oct 20;11:e69725. doi: 10.2196/69725 (PMC12583944; doi:10.2196/69725)
Supplement: Multimedia Appendix 5 [file mededu_v11i1e69725_app5.docx]

## Multimedia Appendix 5

Sanity Checks of the EEG Acquisition System.

### Data quality assessment of the acquisition systems.

To verify the data quality and integrity of the OpenBCI acquisition system, the local Unix timestamp module, and our in-house EEG data streaming software, we conducted 2 additional control experiments. For practical reasons, these experiments were performed on 3 sessions randomly selected from a total of 30 sessions. In the first experiment, we compared resting EEG between cross-fixation eyes-open (EO) and eyes-closed (EC) conditions, with each condition lasting 2 minutes. Figure S1A displayed the EEG power spectra averaged across all eligible participants at each electrode location, comparing EO and EC resting states with (VR) and without (non-VR) the VR headset. In both VR and non-VR conditions, the EC resting state consistently showed a larger alpha rhythm (around 10 Hz) than the EO resting state, aligning with the well-known phenomenon of "alpha blocking" by sensory stimulation [1] .

In the second experiment, participants completed an auditory odd-ball paradigm [2] using custom C# software to deliver two sinus tones (250 Hz and 1000 Hz) via the *Console.Beep* function. Hundred targets (1000 Hz) and 100 non-targets (250 Hz) tones were presented with equal probability, each lasting 175 milliseconds, and separated by random intervals between 900 milliseconds and 1100 milliseconds. The tones were delivered through an external speaker at 70 dB average loudness at each participant's location. During the experiment, participants fixated on a cross at the screen’s center (non-VR condition) or on a virtual object (VR condition), following Thaler et al. [3] with visualizations created in MATLAB using Psychtoolbox [4]. For each target tone, participants clicked the left mouse button as quickly as possible. EEG signals were segmented into epochs from 200 milliseconds before to 800 milliseconds after each tone’s onset, and only correct responses were used for evoke-related potential (ERP) analysis. Figure S1B presents ERP responses, filtered with a 0.05–40 Hz bandpass (using the *pop_eegfiltnew* function in EEGLAB), averaged across eligible participants at Fz, Cz, and Pz for VR and non-VR conditions. For both conditions, clear P200 and P300 responses were observed for the target. Figure S1C shows ERP responses for both tones averaged across VR and non-VR conditions. The mean latency of P200 for the target tone (1000 Hz) was 168.9 milliseconds (SD 13.8 milliseconds), and for the non-target tone (250 Hz), it was 182.6 milliseconds (SD 14.5 milliseconds). A paired-sample *t*-test in SPSS revealed a statistically significant difference in mean latency (*t*(16)=3.195, *P* value<.01), with the target P200 (1000 Hz) showing a shorter mean latency than the non-target P200 (250 Hz). These results align with previous findings [5] that the higher the frequency of the tones below 1000 Hz, the faster the ERP responses.


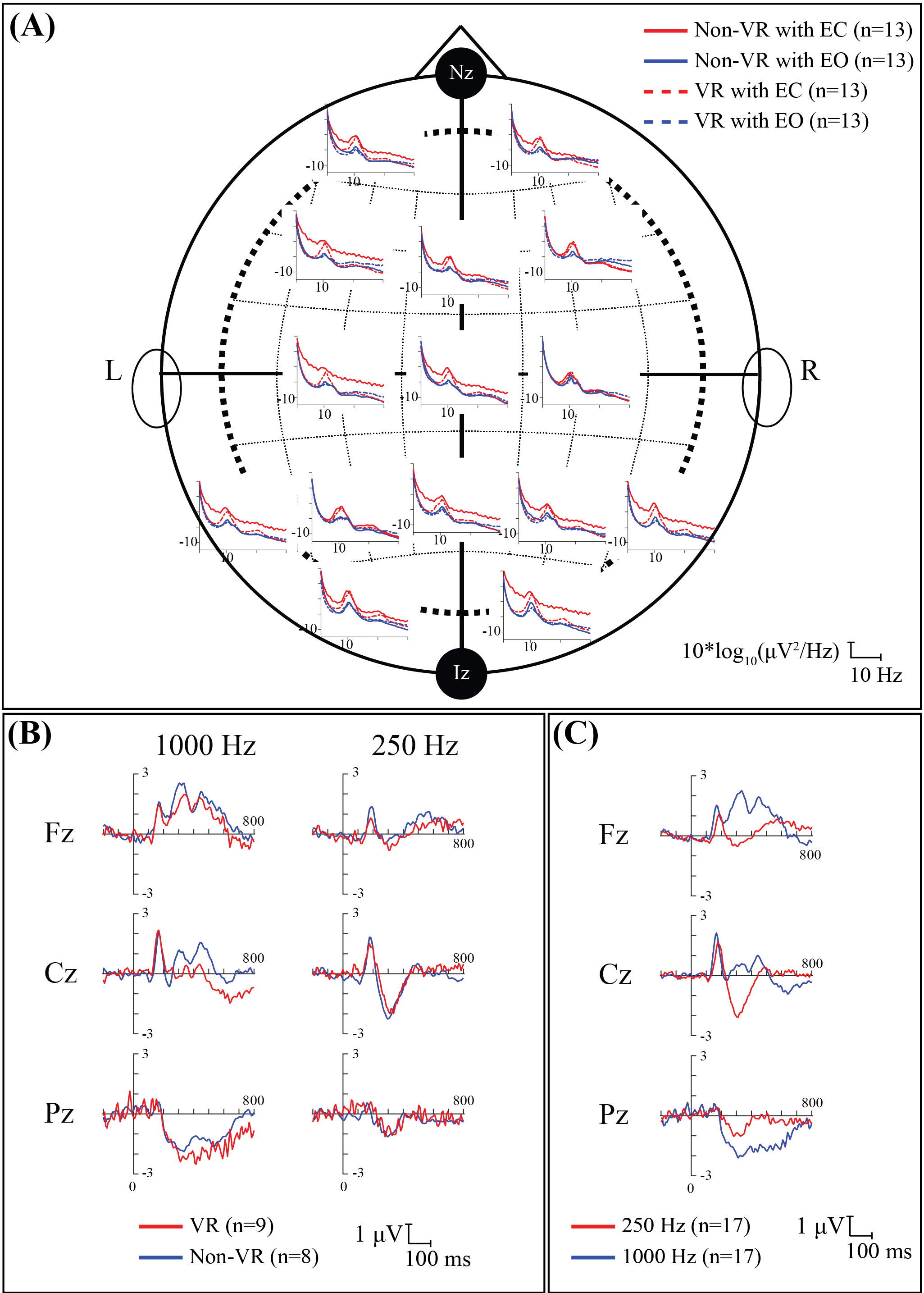


**Figure S1. Power spectrum across testing conditions.** This figure presents the power spectrum data from the control experiments. Panel A displays the average power spectra at each electrode, comparing eyes-open (EO; blue) versus eyes-closed (EC; red) conditions with (dashed line) and without (solid line) the VR headset across 30 sampled participants. Spectra are calculated from 2-minute EEG recordings. During the EO condition, participants fixated on a cross on their screen. Panel B shows event-related potentials (ERPs) in response to 250 Hz (right) and 1000 Hz (left) tones at electrodes Fz (top), Cz (middle), and Pz (bottom), with the VR headset (red) and without the VR headset (blue). Panel C displays combined ERPs for both VR and non-VR conditions, facilitating comparison between the two frequencies. **Abbreviations**: Cz, Central midline; EC, An eye-closed resting condition; EO, An eye-open resting condition; ERP, Event-related potential; Fz, Frontal midline; Iz, Inion; L, Left; Nz, Nasion; Pz, Parietal midline; R, Right; VR, Virtual reality.

### Validating normalized TI as a measure of shared stimulus entrainment.

We calculated the non-normalized group TI for 5 groups of 6 participants engaged in the tone experiments (250 Hz and 1000 Hz) as a function of time after stimulus onset (Figures S2A, top and bottom, respectively). The computed TI represented the average of the anterior, central, and posterior TIs. As shown in Figure S2A, group TI peaked between 300 and 400 milliseconds after the stimulus onset, aligning with ERP components typically observed in the auditory odd-ball paradigm within 400 milliseconds, such as N100-P200-N200-P300 [6, 7]. In Figure S2B, we plotted the non-normalized group TIs at 400 milliseconds alongside their empirical distributions, where the non-normalized group TIs (vertical lines) were clearly distinct from their empirical distributions. These results indicated that normalized group TIs effectively represent shared stimulus entrainment within a group.


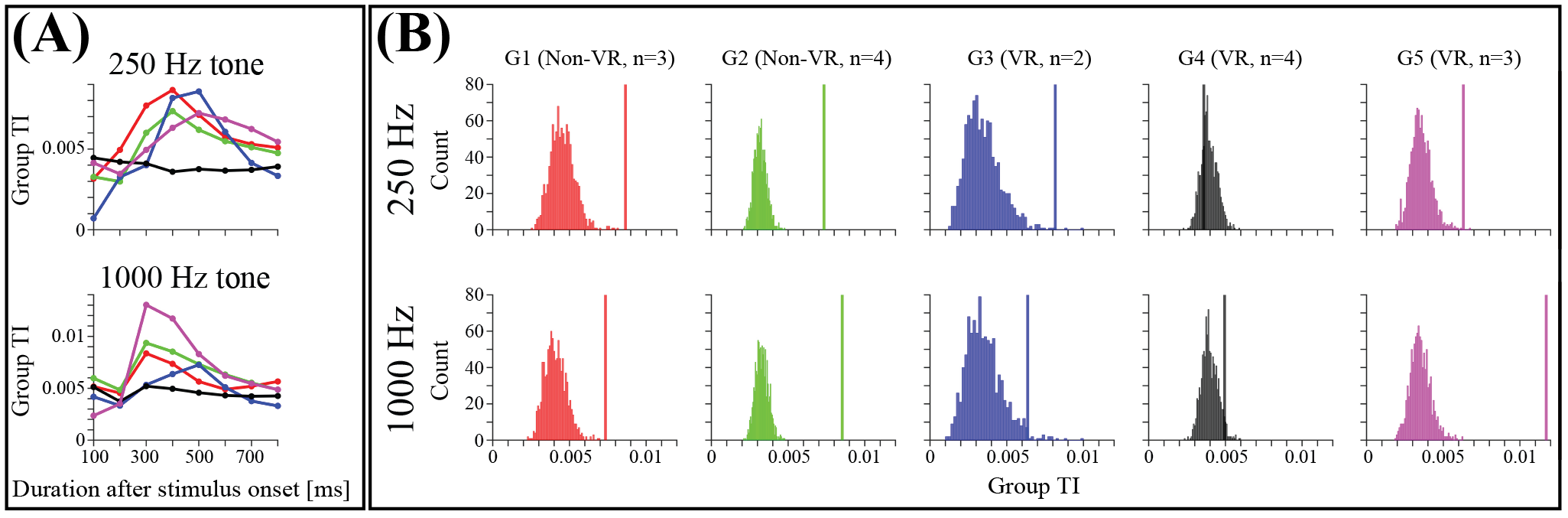


**Figure S2. Validation of normalized TI as a measure of shared stimulus entrainment.** Panel A shows the non-normalized group TIs over time following stimulus onset for the 250 Hz tone (top) and 1000 Hz tone (bottom) across 5 groups, each labeled in Panel B with matching colors. Based on Panel A results, Panel B presents the non-normalized group TIs at 400 milliseconds alongside their empirical distributions. **Abbreviations**: TI, Total interdependence; VR, Virtual reality.

## References

1. Barlow JS. Some aspects of alpha activity in relation to a new generalized model for the eeg. Int J Psychophysiol. 1997 Jun;26(1):341-52. doi: 10.1016/S0167-8760(97)00774-5.

2. Näätänen R, Paavilainen P, Rinne T, Alho K. The mismatch negativity (mmn) in basic research of central auditory processing: A review. Clin Neurophysiol. 2007 Dec;118(12):2544-90. PMID: 17931964. doi: 10.1016/j.clinph.2007.04.026.

3. Thaler L, Schütz AC, Goodale MA, Gegenfurtner KR. What is the best fixation target? The effect of target shape on stability of fixational eye movements. Vision Res. 2013 Jan;76:31-42. doi: 10.1016/j.visres.2012.10.012.

4. Kleiner M, Brainard D, Pelli D. What's new in psychtoolbox-3? Perception. 2007;36(ECVP Abstract Supplement), 14.

5. Roberts TP, Poeppel D. Latency of auditory evoked m100 as a function of tone frequency. Neuroreport. 1996 Apr 26;7(6):1138-40. PMID: 8817518. doi: 10.1097/00001756-199604260-00007.

6. Kappenman ES, Luck SJ. The oxford handbook of event-related potential components. Kappenman ES, Luck SJ, editors: Oxford University Press; 2011 18 Sep 2012. ISBN: 9780195374148.

7. Ochoa CJ, Polich J. P300 and blink instructions. Clin Neurophysiol. 2000 Jan;111(1):93-8. doi: 10.1016/S1388-2457(99)00209-6.
