# Supplementary material for: Correlation Between Electroencephalogram Brain-to-Brain Synchronization and Team Strategies and Tools to Enhance Performance and Patient Safety Scores During Online Hexad Virtual Simulation-Based Interprofessional Education: Cross-Sectional Correlational Study
Source: JMIR Med Educ. 2025 Oct 20;11:e69725. doi: 10.2196/69725 (PMC12583944; doi:10.2196/69725)
Supplement: Multimedia Appendix 6 [file mededu_v11i1e69725_app6.docx]

## Multimedia Appendix 6

EEG Preprocessing

To ensure high quality of the electroencephalogram (EEG) data, we carefully cleaned various artifacts [1] from the signals before further analysis. A flowchart of EEG preprocessing was summarized in Figure S1A. First, EEG data were converted from our proprietary format to BDF format (see Figure S1B left; [2]) for offline preprocessing using EEGLAB software (version 2021; [3]) in MATLAB (version 9.11.0.1809720; [4]). We started the preprocessing by removing 50 Hz line noise (FIR filter using the *pop_eegfiltnew* function of EEGLAB with 47.5 Hz and 52.5 Hz cutoff values) and its 25 Hz sub-harmonic noise (*ZapLine*, version 1.2.1, [5, 6]; *CleanLine*, version 2.0, [7]). We applied both ZapLine and CleanLine to maximally clean the 25 Hz sub-harmonic noise [8]. We then checked for electrode bridging with the *eBridge* EEGLAB plug-in (version 0.1.01; [9]) and marked any bridged electrodes for later removal (mean 0.07, SD 0.43; range 0-4 bridged electrodes). With help from the *Clean Rawdata* EEGLAB plug-in (version 2.7; [10, 11]), electrodes contaminated by excessive noise or other artifacts were manually identified and removed (mean 1.1, SD 1.3; range 0-5 contaminated electrodes), with spherical interpolation (the *pop_interp* function of EEGLAB) used to replace the missing data (mean 1.1, SD 1.4; range 0-6 interpolated electrodes). Once cleaned, the EEG signals were re-referenced to the average reference [12]. Finally, the data (see the processed raw EEG signal in Figure S1B) were split into two copies: one for independent component analysis (ICA; see Figure S1A right branch; [13]) and the other for the total interdependence (TI) analysis (see Figure S1A left branch; [14, 15]).


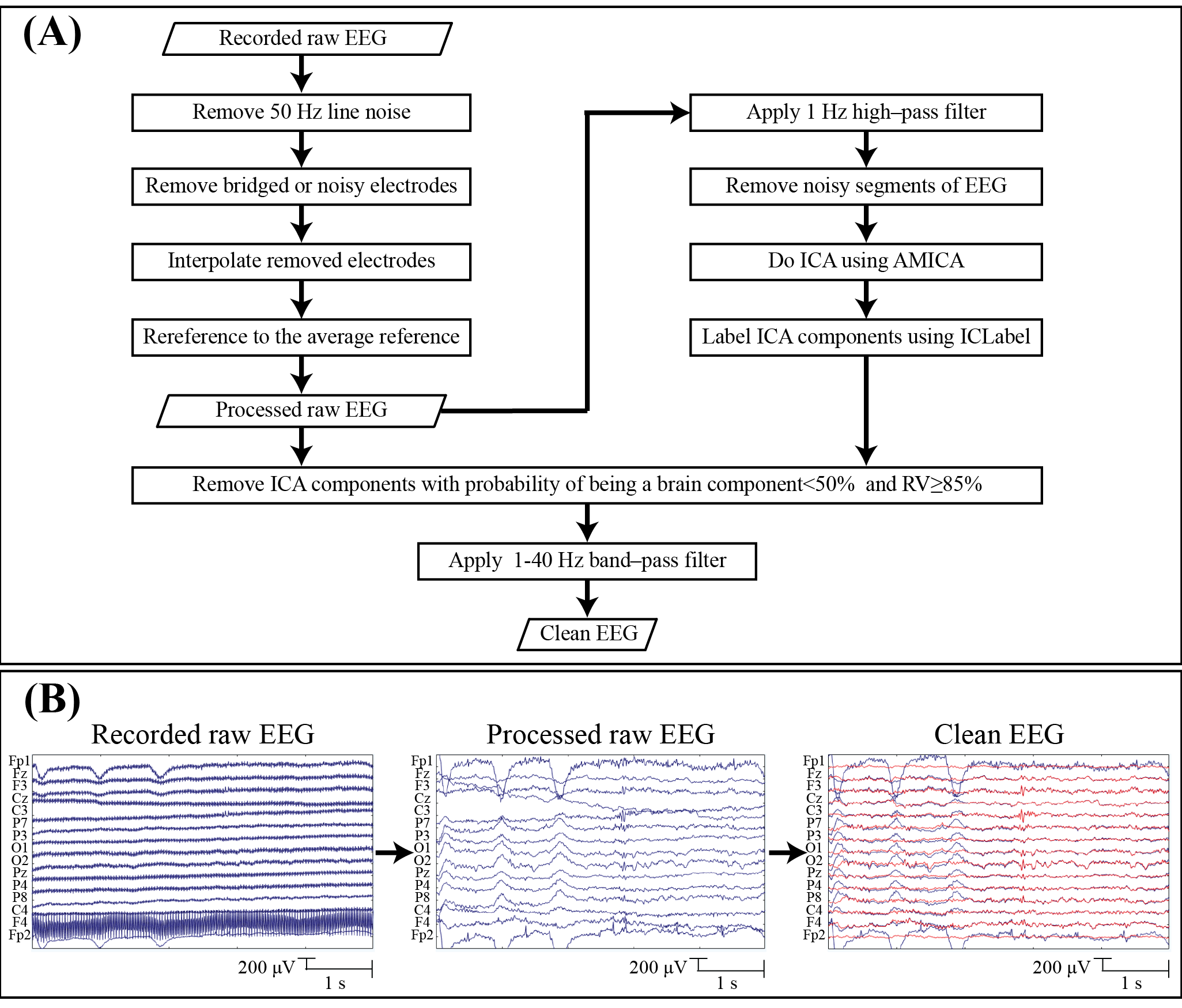


**Figure S1. EEG preprocessing schematic.** Panel A shows steps of EEG preprocessing, in which ICA components obtained from the ICA analysis (right branch) are used for cleaning the EEG signals (left branch). This results in the clean EEG signals for the TI analysis. Panel B demonstrates an example of the EEG signal before preprocessing (left), after the initial steps (middle), and after removing ICA artifact components (right; red). **Abbreviations**: AMICA, Adaptive mixture independent component analysis; EEG, Electroencephalogram; ICA, Independent component analysis; RV, Residual variance.

The first copy of the EEG signal was applied with a high-pass filter at 1 Hz to remove low-frequency noise (FIR filters using the *pop_eegfiltnew* function of EEGLAB). Since ICA is sensitive to infrequent atypical artifacts [1, 16], we manually inspected and removed any contaminated segments (14.87%, SD 16.91%; range 0.04-87.35% of the EEG signal; the *eeg_eegrej* and *pop_eegplot* functions of EEGLAB). ICA was then performed using the AMICA algorithm (version 1.6; [17]) with a principal component analysis rank reduction, which was set to the number of electrodes (i.e. 15) minus one (the average reference) minus the number of interpolated electrodes [1, 18]. We classified the ICA components using the *ICLabel* EEGLAB plug-in (version 1.4; [19]), marking those with the residual variance above 85% (80.77%, SD 12.77%; range 42.86-100% of ICA components; [20]) and the brain probability below 50% (30.82%, SD 11.38%; range 14.29-72.73% of ICA components; [21]) for rejection. After removing these components from the second copy of the EEG signal (the *pop_subcomp* function of EEGLAB), we then applied a bandpass filter (1-40 Hz) to it to obtain a clean EEG signal (see the red curves in Figure S1B right). To improve the signal-to-noise ratio, we grouped the electrodes into anterior, central, and posterior brain regions, averaging signals within each group to reduce dimensionality from 15 to 3 channels for further TI analysis. Finally, the clean EEG signal remaining after removing the artifact segments was used to compute 3 golden SDs, each representing the non-biased standard deviation of the 3 averages.

## References

1. Klug M, Gramann K. Identifying key factors for improving ica-based decomposition of eeg data in mobile and stationary experiments. Eur J Neurosci. 2020;54(12). doi: 10.1111/ejn.14992.

2. BIOSEMI. What is the .Bdf file format? (biosemi data format). 2023; <https://www.biosemi.com/faq/file_format.htm> [accessed December 22, 2023].

3. Delorme A, Makeig S. Eeglab: An open source toolbox for analysis of single-trial eeg dynamics including independent component analysis. J Neurosci Methods. 2004 Mar 15;134(1):9-21. PMID: 15102499. doi: 10.1016/j.jneumeth.2003.10.009.

4. Inc. TM. Matlab version: 9.11.0.1809720 (r2021b) update 1. 2021; <https://www.mathworks.com> [accessed December 22, 2023].

5. de Cheveigné A. Zapline: A simple and effective method to remove power line artifacts. Neuroimage. 2020 Feb;207:116356. doi: 10.1016/j.neuroimage.2019.116356.

6. Klug M, Kloosterman NA. Zapline-plus: A zapline extension for automatic and adaptive removal of frequency-specific noise artifacts in m/eeg. Hum Brain Mapp. 2022;43(9):2743-58. doi: 10.1002/hbm.25832.

7. Mullen T. Cleanline. 2012; <https://www.nitrc.org/projects/cleanline> [accessed November 15, 2023].

8. Miyakoshi M, Schmitt LM, Erickson CA, Sweeney JA, Pedapati EV. Can we push the "quasi-perfect artifact rejection" even closer to perfection? Front Neuroinform. 2020;14:597079. PMID: 33584237. doi: 10.3389/fninf.2020.597079.

9. Alschuler DM, Tenke CE, Bruder GE, Kayser J. Identifying electrode bridging from electrical distance distributions: A survey of publicly-available eeg data using a new method. Clin Neurophysiol. 2014;125(3):484-90. doi: 10.1016/j.clinph.2013.08.024.

10. Kothe CA, Makeig S. Bcilab: A platform for brain-computer interface development. J Neural Eng. 2013 Oct;10(5):056014. PMID: 23985960. doi: 10.1088/1741-2560/10/5/056014.

11. Miyakoshi M, Jurgiel J, Dillon A, Chang S, Piacentini J, Makeig S, et al. Modulation of frontal oscillatory power during blink suppression in children: Effects of premonitory urge and reward. Cereb Cortex Commun. 2020;1(1). doi: 10.1093/texcom/tgaa046.

12. Dien J. Issues in the application of the average reference: Review, critiques, and recommendations. Behav Res Methods Instrum Comput. 1998 Mar;30(1):34-43. doi: 10.3758/bf03209414.

13. Makeig S, Bell AJ, Jung T-P, Sejnowski TJ. Independent component analysis of electroencephalographic data. Proceedings of the 8th International Conference on Neural Information Processing Systems; Denver, Colorado: MIT Press; 1995. p. 145–51.

14. Gel’fand IM, Yaglom AM. Calculation of the amount of information about a random function contained in another such function: American Mathematical Society Translations: Series 2; 1959.

15. Geweke J. Measurement of linear dependence and feedback between multiple time series. J Am Stat Assoc. 1982;77(378):304-13. doi: 10.1080/01621459.1982.10477803.

16. Winkler I, Haufe S, Tangermann M. Automatic classification of artifactual ica-components for artifact removal in eeg signals. Behav Brain Funct. 2011 Aug;7(1):30. doi: 10.1186/1744-9081-7-30.

17. Delorme A, Palmer J, Onton J, Oostenveld R, Makeig S. Independent eeg sources are dipolar. PLOS ONE. 2012;7(2):e30135. doi: 10.1371/journal.pone.0030135.

18. Hyvärinen A, Oja E. Independent component analysis: Algorithms and applications. Neural Netw. 2000;13(4-5):411-30. doi: 10.1016/S0893-6080(00)00026-5.

19. Pion-Tonachini L, Kreutz-Delgado K, Makeig S. Iclabel: An automated electroencephalographic independent component classifier, dataset, and website. Neuroimage. 2019 Sep;198:181-97. doi: 10.1016/j.neuroimage.2019.05.026.

20. Onton J, Westerfield M, Townsend J, Makeig S. Imaging human eeg dynamics using independent component analysis. Neurosci Biobehav Rev. 2006;30(6):808-22. doi: 10.1016/j.neubiorev.2006.06.007.

21. Arnau S, Löffler C, Rummel J, Hagemann D, Wascher E, Schubert A-L. The electrophysiological signature of mind wandering. bioRxiv. Preprint posted online October 25, 2019. doi: 10.1101/819805.
