# Supplementary material for: Correlation Between Electroencephalogram Brain-to-Brain Synchronization and Team Strategies and Tools to Enhance Performance and Patient Safety Scores During Online Hexad Virtual Simulation-Based Interprofessional Education: Cross-Sectional Correlational Study
Source: JMIR Med Educ. 2025 Oct 20;11:e69725. doi: 10.2196/69725 (PMC12583944; doi:10.2196/69725)
Supplement: Multimedia Appendix 7 [file mededu_v11i1e69725_app7.docx]

## Multimedia Appendix 7

Correlation between Student TI and the TeamSTEPPS Scores.


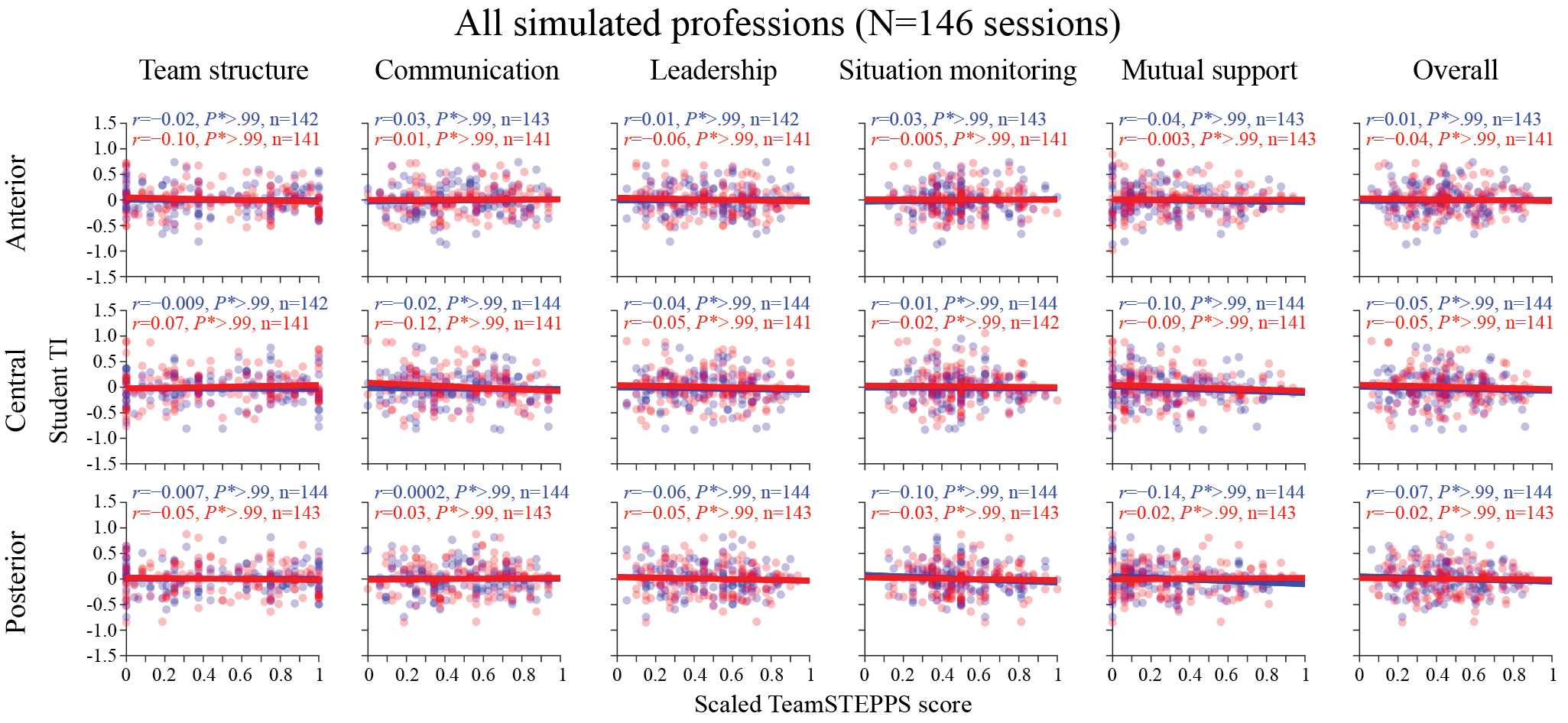


**Figure S1. No correlation between the student TI and the TeamSTEPPS scores.** Each scatter plot illustrates the relationship between the student TIs and the individual scaled TeamSTEPPS scores for the anterior (top row), central (second row), and posterior (third row) brain areas, across all frequency bands (blue) and the alpha band (red). Each column represents results for individual TeamSTEPPS domains, with the final column showing the total score across all domains. The legend in each plot provides the Pearson correlation coefficient (*r*), the adjusted *P* value (*P**), and the sample size after excluding outliers (n). The straight lines represent the best fit according to the least-squares method. The figure indicates no statistically significant correlation between the student TI and the individual scaled TeamSTEPPS scores (*P**≥.05). **Abbreviations**: TI, Total interdependence.


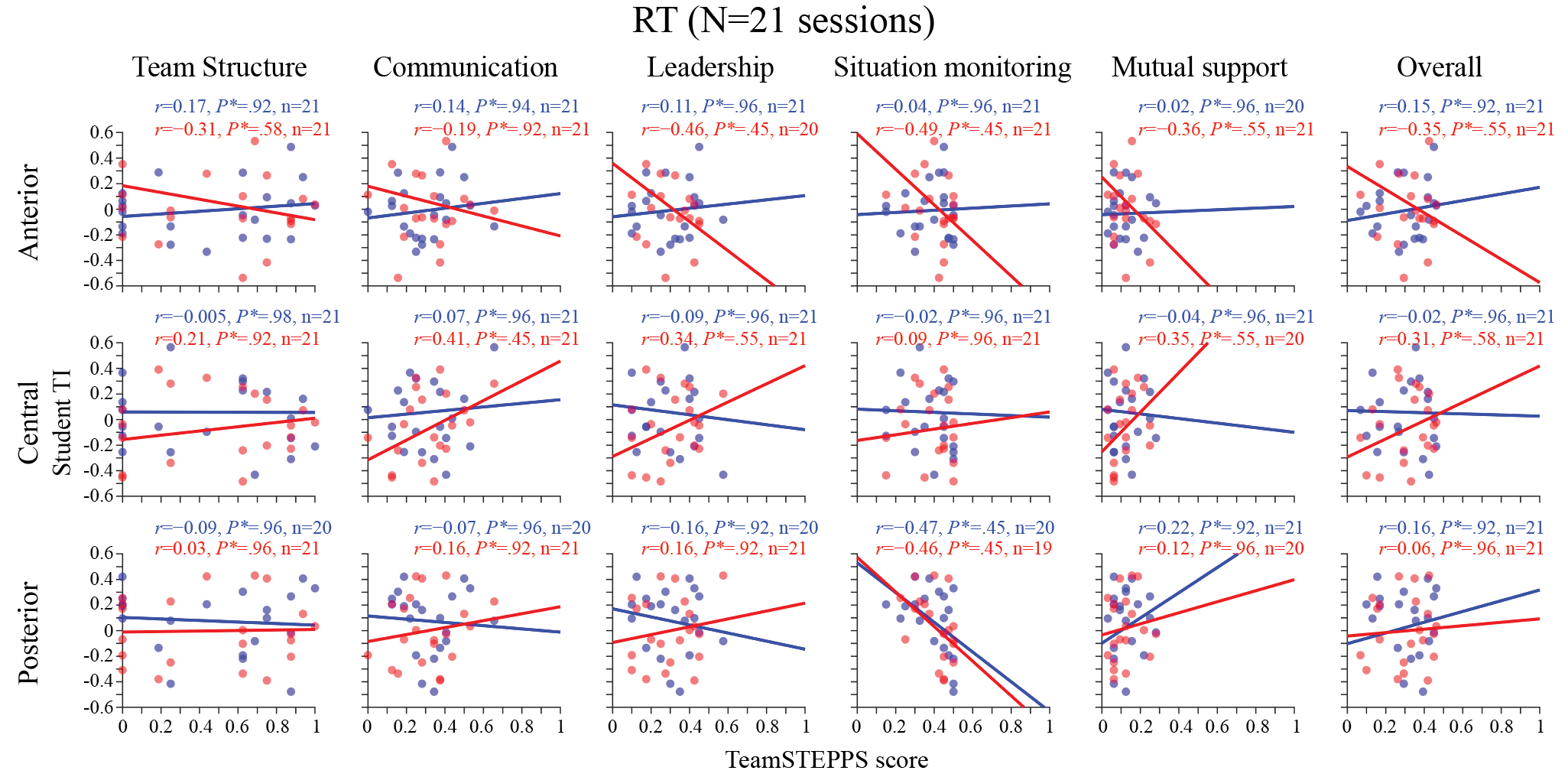


**Figure S2.** **No correlation between TIs of the RT students and their TeamSTEPPS scores.** Each scatter plot illustrates the relationship between the student TIs of the RT students and the individual scaled TeamSTEPPS scores for the anterior (top row), central (second row), and posterior (third row) brain areas, across all frequency bands (blue) and the alpha band (red). Indicated by *P**≥.05, figure shows no statistically significant correlation between the student TI and the scaled TeamSTEPPS score for RT profession. Note: *P** represented the adjusted *P* value. **Abbreviations**: RT, Radiological technologist; TI, Total interdependence.


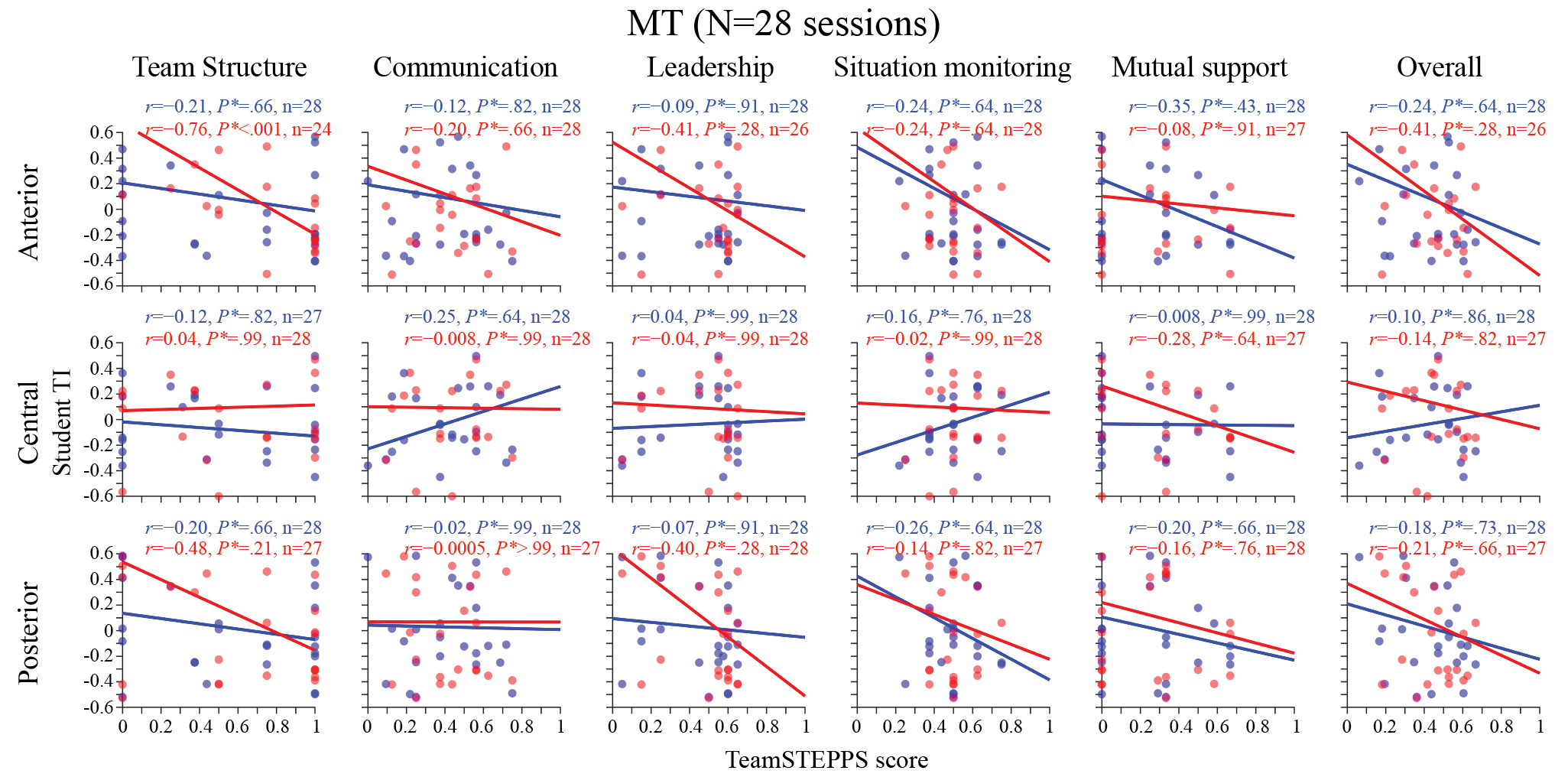


**Figure S3.** **No correlation between TIs of the MT studen**t**s and their TeamSTEPPS scores.** Each scatter plot illustrates the relationship between the student TIs of the MT students and the individual scaled TeamSTEPPS scores for the anterior (top row), central (second row), and posterior (third row) brain areas, across all frequency bands (blue) and the alpha band (red). Indicated by *P**≥.05, figure shows no statistically significant correlation between the student TI and the scaled TeamSTEPPS score for MT profession, except the team structure topic in the anterior brain area (*P**<.001). Note: *P** represented the adjusted *P* value. **Abbreviations**: MT, Medical technologist; TI, Total interdependence.


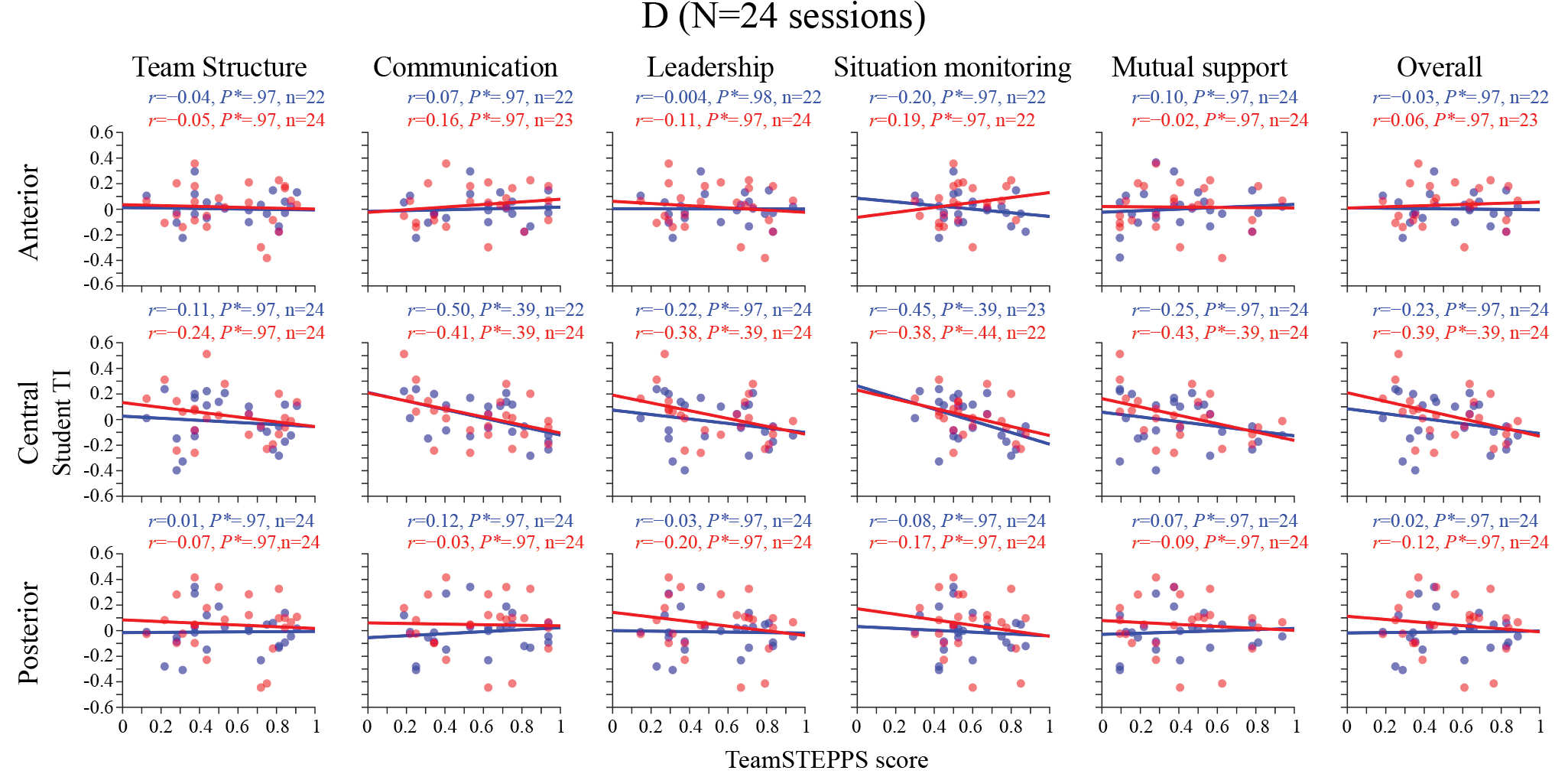


**Figure S4.** **No correlation between TIs of the D students and their TeamSTEPPS scores.** Each scatter plot illustrates the relationship between the student TIs of the D students and the individual scaled TeamSTEPPS scores for the anterior (top row), central (second row), and posterior (third row) brain areas, across all frequency bands (blue) and the alpha band (red). Indicated by *P**≥.05, figure shows no statistically significant correlation between the student TI and the scaled TeamSTEPPS score for D profession. Note: *P** represented the adjusted *P* value. **Abbreviations**: D, Medical doctor; TI, Total interdependence.


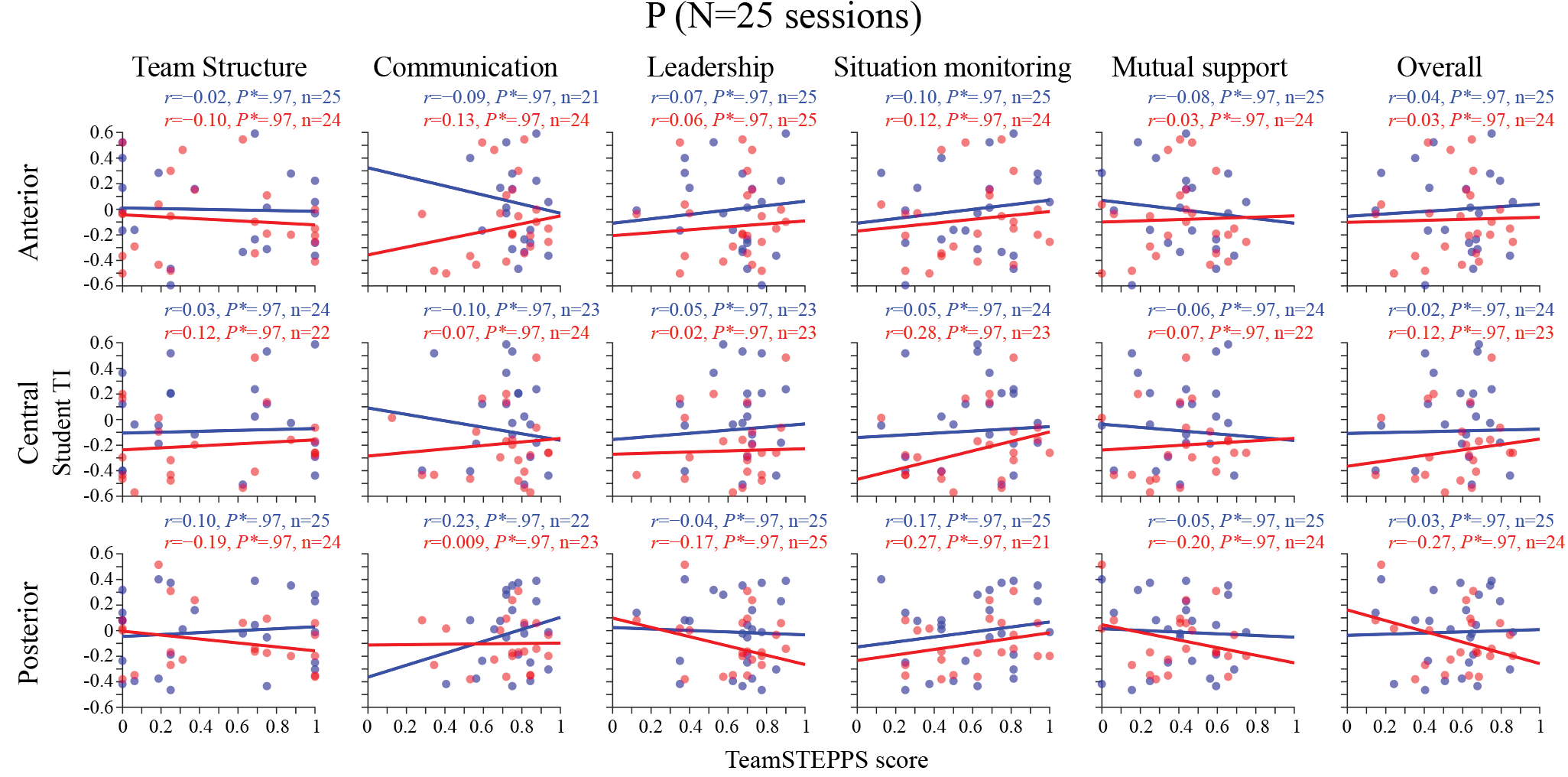


**Figure S5. No correlation between TIs of the P students and their TeamSTEPPS scores.** Each scatter plot illustrates the relationship between the student TIs of the P students and the individual scaled TeamSTEPPS scores for the anterior (top row), central (second row), and posterior (third row) brain areas, across all frequency bands (blue) and the alpha band (red). Indicated by *P**≥.05, figure shows no statistically significant correlation between the student TI and the scaled TeamSTEPPS score for P profession. Note: *P** represented the adjusted *P* value. **Abbreviations**: P, Pharmacist; TI, Total interdependence.


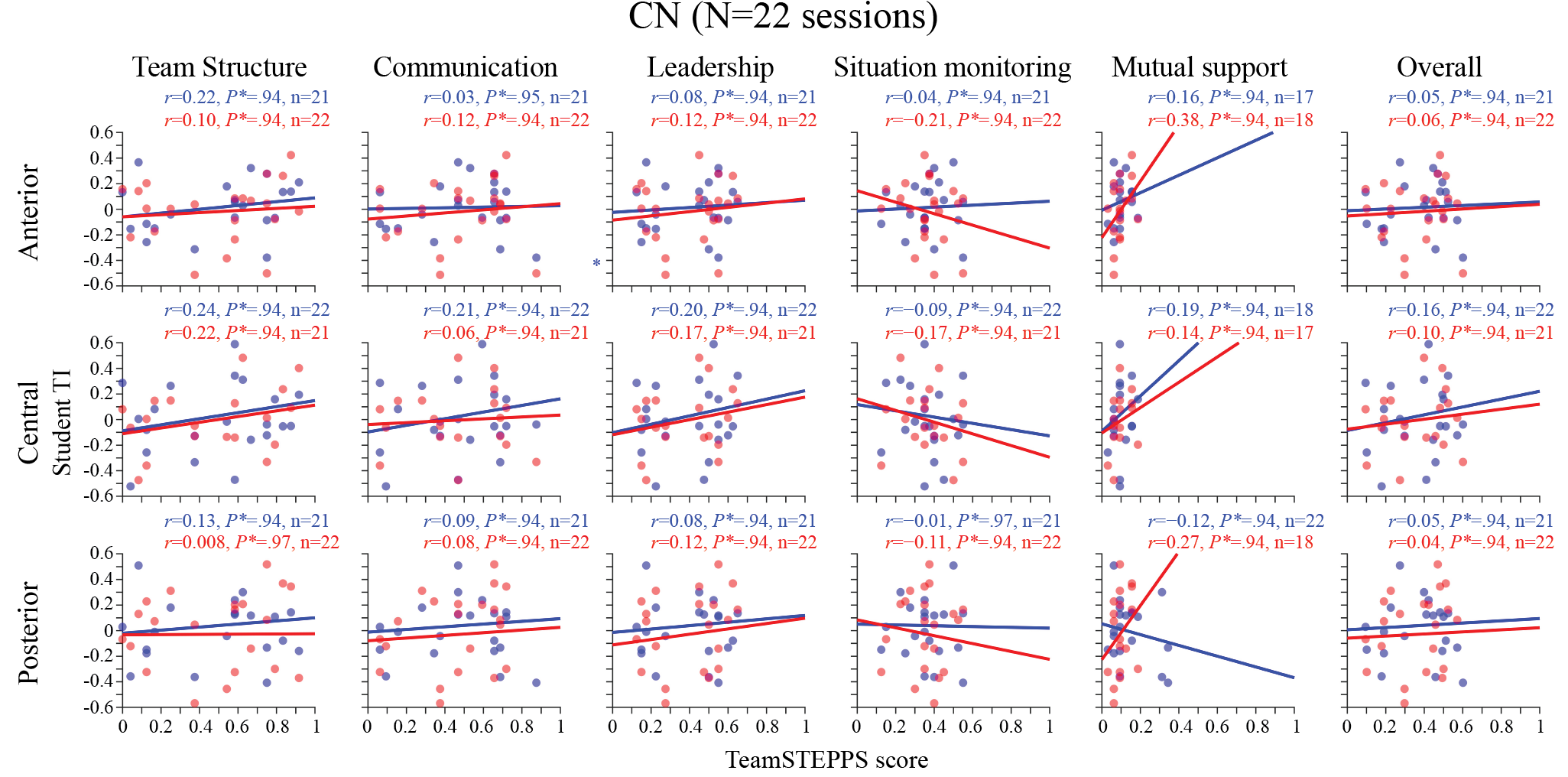


**Figure S6.** **No correlation between TIs of the CN students and their TeamSTEPPS scores.** Each scatter plot illustrates the relationship between the student TIs of the CN students and the individual scaled TeamSTEPPS scores for the anterior (top row), central (second row), and posterior (third row) brain areas, across all frequency bands (blue) and the alpha band (red). Indicated by *P**≥.05, figure shows no statistically significant correlation between the student TI and the scaled TeamSTEPPS score for CN profession. Note: *P** represented the adjusted *P* value. **Abbreviations**: CN, Circulation nurse; TI, Total interdependence.


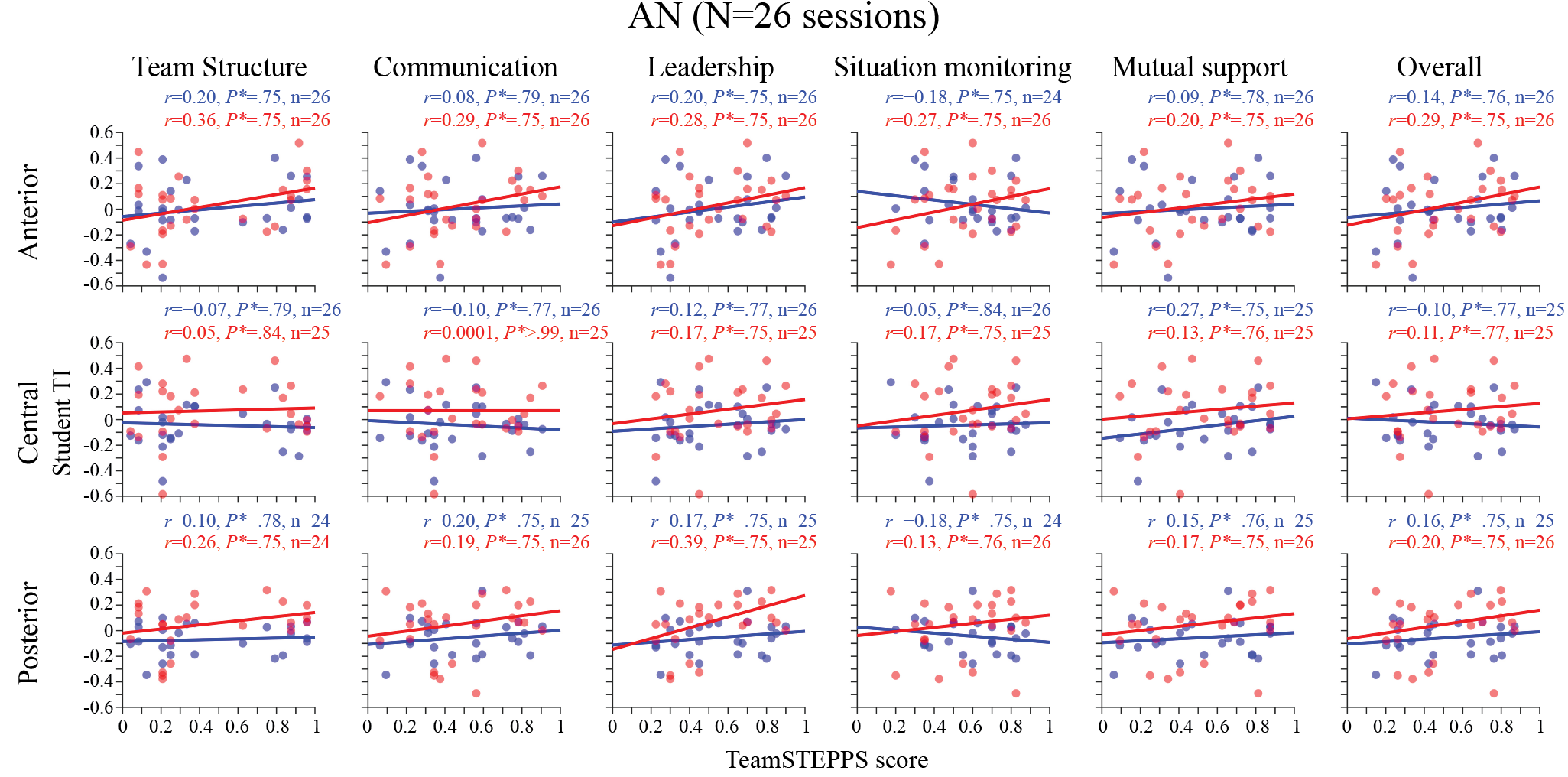


**Figure S7.** **No correlation between TIs of the AN students and their TeamSTEPPS scores.** Each scatter plot illustrates the relationship between the student TIs of the AN students and the individual scaled TeamSTEPPS scores for the anterior (top row), central (second row), and posterior (third row) brain areas, across all frequency bands (blue) and the alpha band (red). Indicated by *P**≥.05, figure shows no statistically significant correlation between the student TI and the scaled TeamSTEPPS score for AN profession. Note: *P** represented the adjusted *P* value. **Abbreviations**: AN, Airway nurse; TI, Total interdependence.
