# Supplementary material for: Correlation Between Electroencephalogram Brain-to-Brain Synchronization and Team Strategies and Tools to Enhance Performance and Patient Safety Scores During Online Hexad Virtual Simulation-Based Interprofessional Education: Cross-Sectional Correlational Study
Source: JMIR Med Educ. 2025 Oct 20;11:e69725. doi: 10.2196/69725 (PMC12583944; doi:10.2196/69725)
Supplement: Multimedia Appendix 8 [file mededu_v11i1e69725_app8.docx]

## Multimedia Appendix 8

Correlation between the Communication TI of a Pair and Characteristics of Communications.

### Duration of communications.

The characteristic that we investigated was the duration of communications.


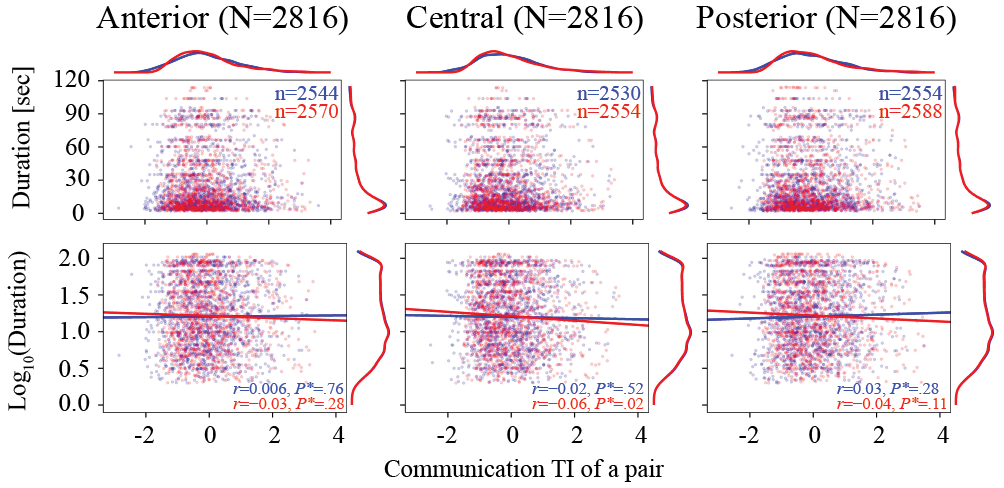


**Figure S1. The communication TI of a pair versus communication duration.** Scatter plots of the communication TI of a pair versus communication duration for the anterior (left), central (middle), and posterior (right) brain areas, are shown before (top) and after (bottom) applying the 10-base logarithmic transformation to communication duration: Blue represents all frequency bands; red represents the alpha band. Histograms of the data are displayed along the top and right edges of each plot. The legends in the bottom scatter plots show the Pearson correlation (*r*) and the adjusted *P* value (*P**), while they show the sample size after excluding outlines (n) in the top plots. No significant correlation was observed between the communication TI of a pair and the communication duration, except for a low-value correlation in the central brain area with the alpha frequency band. **Abbreviations**: TI, Total interdependence.
